# Conventional probe trabeculotomy versus microcatheter-assisted 360° trabeculotomy (PIRATE) in childhood glaucoma—study protocol for a randomized controlled trial

**DOI:** 10.1186/s13063-025-09091-3

**Published:** 2025-09-22

**Authors:** Julia V. Stingl, Irene M. Schmidtmann, Markus Schepers, Alexander K. Schuster, Jasmin Rezapour, Anna M. Welzel, Angi L. Mendoza-Moreira, Heike Elflein, Anne Ehrlich, Claudia Wolf, Michael Hopp, Ingeborg Stalmans, Sophie Lemmens, Thomas Dietlein, Silvia Schrittenlocher, Alexandra Lappas, Claudia Schuart, Hagen Thieme, Esther M. Hoffmann

**Affiliations:** 1 Department of Ophthalmology, University Medical Center of the Johannes Gutenberg-University Mainz, Mainz, Germany; 2 Institute of Medical Biostatistics, Epidemiology and Informatics, University Medical Center of the Johannes Gutenberg-University Mainz, Mainz, Germany; 3 Interdisciplinary Center for Clinical Studies, University Medical Center of the Johannes Gutenberg-University Mainz, Mainz, Germany; 4 Department of Ophthalmology, UZ Leuven, Leuven, Belgium; 5 Research Group Ophthalmology, KU Leuven, Leuven, Belgium; 6 Department of Ophthalmology, Faculty of Medicine and University Hospital Cologne, University of Cologne, Cologne, Germany; 7 Department of Ophthalmology, Medical Faculty of Otto Von Guericke University Magdeburg, Magdeburg, Germany

**Keywords:** Childhood glaucoma, Trabeculotomy, Buphthalmos, Congenital glaucoma, Probe trabeculotomy, Microcatheter-assisted 360° trabeculotomy, Randomized controlled trial

## Abstract

**Background:**

To compare the success and safety of microcatheter-assisted 360° trabeculotomy (MCAT) with conventional probe trabeculotomy in a large, heterogeneous cohort of children with primary or secondary glaucoma.

**Methods:**

In this prospective, multicenter, observer-blinded, randomized controlled trial, 76 children (152 eyes) with bilateral primary or secondary childhood glaucoma aged ≤ 12 years will be included. Each child acts as own control using a paired-eye design: One eye is allocated to MCAT (experimental intervention), achieving a 360° trabeculotomy, the other eye to the probe trabeculotomy (control intervention) which enables a trabeculotomy over 90 to 120°. Each child receives both procedures (paired-eye design). The worse eye is treated first; the surgical method is randomized. Patients and observers are masked to the procedures. The patients are followed up for 24 months. The primary endpoint is complete success (IOP < 18 mmHg at 24 months without medication and revision surgery; with MCAT: successful probing of > 120° is also required for success) at 24 months of follow-up. The primary analysis is performed in the intention-to-treat population using McNemar test stratified by center.

**Discussion:**

The PIRATE study is a multicenter randomized controlled study comparing MCAT with conventional probe trabeculotomy in a large and heterogeneous childhood glaucoma population. It will provide data on the success and safety of both techniques and clarify if MCAT is superior to probe trabeculotomy.

**Trial registration:**

German Clinical Trials Register, DRKS-ID: DRKS00034139. Registered on April 24, 2024. https://drks.de/search/en/trial/DRKS00034139. https://trialsearch.who.int/Trial2.aspx?TrialID=DRKS00034139.

**Supplementary Information:**

The online version contains supplementary material available at 10.1186/s13063-025-09091-3.

## Background

Childhood glaucoma leads to loss of visual function if not treated. The cause is high intraocular pressure (IOP) due to a dysgenesis of the outflow pathway of the aqueous humor. Most cases of congenital glaucoma need surgery [1–3]. Trabeculotomy, which opens Schlemm’s canal and therewith the eye’s drainage system, is the most common surgical intervention and is considered the gold standard [3–6]. In conventional probe trabeculotomy, bowed metal probes are inserted ab externo into the Schlemm’s canal, which is opened by rotating the probe into the anterior chamber [7]. The 1-year success rates of conventional trabeculotomy in primary congenital glaucoma vary from 47% to over 92% depending on glaucoma severity and kind of glaucoma [8, 9]. A second and newer approach is circumferential trabeculotomy, using an illuminated microcatheter for dilatation and opening the Schlemm’s canal over 360°. A circumferential opening of Schlemm’s canal may theoretically lead to a better IOP decrease than a partial opening as performed in probe trabeculotomy.

At the present time, two prospective randomized controlled studies comparing microcatheter-assisted trabeculotomy (MCAT) with conventional probe trabeculotomy [8, 10] have been published. El Sayed and Gawdat reported a complete success (defined as IOP < 18 mmHg, no other signs of glaucoma progression, no antiglaucoma medications) of 67% versus 47% for MCAT compared to conventional probe trabeculotomy after 2-year follow-up in a sample with 62 Egyptian patients (62 eyes) with primary congenital glaucoma [8]. Shakrawal et al. achieved 1-year complete success in 80% versus 60% with stricter criteria for complete success (IOP ≤ 12 mmHg, no antiglaucoma medications) in a sample with 31 Indian patients (40 eyes) with primary congenital glaucoma [10].

The largest retrospective study performed by Berger et al. showed higher complete surgical success (69% versus 23%, *p* < 0.0001) with significantly lower revision rates after MCAT than after conventional trabeculotomy with a rigid probe in 77 children (106 eyes) with glaucoma [11].

Two systematic reviews with meta-analyses (Ling et al. also included three retrospective studies in addition to the two RCTs) showed a lower IOP and a higher probability for complete and qualified success for MCAT than for probe trabeculotomy, but also a higher risk for the occurrence of hyphema [12, 13]. However, there was a high heterogeneity in the intervention groups and a high probability of performance and observation bias due to lack of masking [12, 13].

Although various attempts have been made to evaluate the efficacy and safety of MCAT compared to probe trabeculotomy, sample sizes and the trial quality are low. Only two randomized controlled studies are available, which comprised Egyptian and Indian patients. Furthermore, most trials did not include secondary glaucoma patients. Thus, there is an unmet need for a large-scale, multicenter prospective randomized controlled trial investigating a real-life childhood glaucoma population. The researchers expect similar results as shown in the aforementioned previous studies, with superiority of 360° trabeculotomy compared to conventional probe trabeculotomy (TO). The results of a larger scaled study will contribute to the acceptance of MCAT as a standard therapy for both primary and secondary childhood glaucoma patients.

## Methods

### Aim, design and study setting

The aim of this study is to compare microcatheter-assisted 360° trabeculotomy with conventional probe trabeculotomy in children aged ≤ 12 years with primary or secondary glaucoma.

The primary hypothesis of this study is that MCAT is superior to probe trabeculotomy with respect to complete success at 24 months after surgery. The key secondary hypothesis is that MCAT is superior to probe trabeculotomy with respect to incomplete success at 24 months after surgery. Further secondary hypotheses address the superiority of MCAT with respect to complete and incomplete success at earlier time points and the superiority of MCAT with respect to other clinical parameters.

Four academic centers are contributing to the study. The coordinating center is the Department of Ophthalmology, University Medical Center Mainz (Germany), which is an academic hospital. Further centers are the Department of Ophthalmology, University Clinic Cologne (Germany), the Department of Ophthalmology, University Hospital Magdeburg (Germany), and the Department of Ophthalmology, UZ Leuven (Belgium).

A paired-eye design is used: each patient receives both interventions (experimental intervention in one eye and control intervention in the other eye in randomized sequence) and acts as his or her own control thus minimizing the influence of external factors on the outcome and the case numbers. The worse affected eye (i.e., higher IOP, longer axial length, greater corneal diameters, and greater cup-to-disc ratio) will be treated first; the investigator assesses which eye is worse affected.

To prevent selection bias, intervention sequence (probe TO, MCAT) will be randomized. Block randomization will be applied using variable block length and stratification by center. Sealed numbered envelopes generated by the coordinating center are provided containing which surgical technique must be used on the first and second eye, respectively.

To prevent observation bias, an independent observer masked to the intervention and not involved in the surgery will conduct the follow-up visits. At the slit lamp, a morphological differentiation between MCAT and probe TO is not possible and postsurgical treatments are the same after both procedures, so making this easily possible. Furthermore, patients, their parents, and their treating pediatricians and ophthalmologists are also masked to the intervention.

**Fig. 1:**
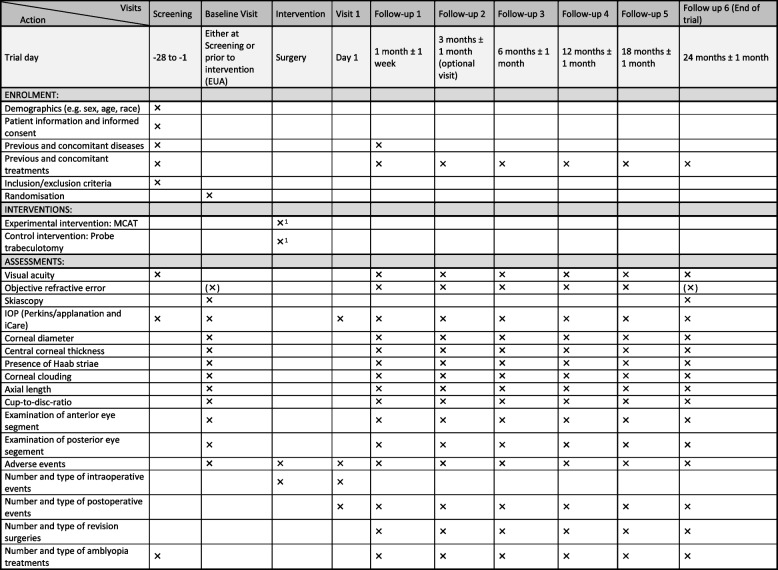
SPIRIT figure presenting the time schedule of enrollment, interventions, and assessments. Additional visits for clinical reasons will also be documented. Abbreviations: IOP, intraocular pressure; EUA, examination under anesthesia; AE, adverse event. ^1^Bilateral surgery should be preferred if clinically feasible; if not, sequential surgery can be performed. ^2^Visit 1 and follow-up visits refer to the date of surgery per eye

The patients will be followed up for 24 months. Six follow-up visits are planned (after 1, 3, 6, 12, 18, and 24 months), where the 3-month follow-up visit is optional. The follow-up visits adhere to the standard of clinical care and are well accepted by the patients and caregivers. Thus, complete follow-up is very likely. Additional examinations, which will be performed for a clinical reason, will also be documented. Examinations take place either in awake setting or under general anesthesia (examination under anesthesia, EUA), depending on the cooperation of the child and on the discretion of the observer. The full study schedule is presented in Fig. 1. In case of withdrawal from the study, an end-of-trial examination is performed.

An interim analysis will be conducted when 38 patients (half of the initially planned sample) have had their 6-month follow-up visit. The interim analysis is conducted because the correlation between the successes of the two eyes of a patient is unknown. The interim analysis to be performed by the data safety monitoring board (DSMB) only has the aim to obtain a better proxy of the correlation and hence the proportion of discordant pairs which impact the required sample size for the desired significance level and power. Simulations performed by one of the study statisticians indicate that the significance level of the final analysis will be kept after this interim analysis. As the primary endpoint will be determined 2 years after treatment, the only possible change in study design is an increase in sample size (up to a maximum of 83 rather than 76). Neither stop for futility nor early stop for success is planned.

### Participants

One hundred fifty-two eyes of 76 children with bilateral primary or secondary childhood glaucoma will be included. Inclusion and exclusion criteria are as follows:

- Inclusion criteria
  - Ability of participant’s legal representatives to understand nature, importance, and individual consequences of clinical trial.
  - Signed and dated informed consent of legal representatives and children, if applicable, must be available before start of any specific trial procedures. Both caregivers must sign the informed consent.
  - Patients with primary congenital or different types of secondary childhood glaucoma at age ≤ 12 years with both eyes requiring trabeculotomy and no other previous glaucoma surgeries.
- Exclusion criteria
  - Indication other than trabeculotomy, e.g., glaucoma drainage device implantation or cyclodestructive treatment
  - Prior glaucoma surgery (other surgeries, such as lensectomy or vitrectomy, are allowed)
  - Language barrier
  - Medical or psychological conditions that would jeopardize an adequate and orderly completion of the trial

### Interventions

#### Microcatheter-assisted 360° trabeculotomy (MCAT)

A limbal-based conjunctival peritomy at 6 mm from the limbus is performed. Then, a partial-thickness scleral flap of size 4 × 4 mm is created. The second, deep scleral flap of size 1.5 × 3 mm is created within the borders of the superficial flap dissecting forward, exposing the choroid, into clear cornea. The roof of Schlemm’s canal (SC) is carefully detached with multifunctional 25-gauge forceps. Then, insertion of an illuminated microcatheter is performed over 360°. Every 60°, viscoelastic is injected into SC via the screw-driven injector. The optical fiber that illuminates the tip of the microcatheter provides guidance to the path of the catheter as it is advanced. Care is taken to keep the catheter in perpetual motion through SC when viscoelastic is injected to prevent the creation of a Descemet’s membrane detachment. When the distal tip of the catheter re-emerges from the opposite opening of SC, a paracentesis wound is created with a 15-degree side port knife at the temporal clear cornea. Acetylcholine chloride intraocular solution is given to constrict the pupil. Viscoelastic to protect lens and endothelium is injected. Then, both ends of the catheter are pulled to create a circumferential rupture of the trabecular meshwork (TM). If probing over 360° is not possible, a scleral incision at the site of the catheter’s tip is performed and the catheter is emerged at this point, and the catheter ends are pulled to create the TM rupture (probing of at least 120° is necessary to successfully conduct the rupturing). The scleral flap and conjunctiva are sutured tightly and viscoelastic is removed from the anterior chamber. Subconjunctival injection of 4 mg dexamethasone is performed in the inferior fornix of the conjunctiva, and then antibiotic ointment is applied prior to bandage.

#### Conventional probe trabeculotomy

Similar opening for both surgeries. After de-roofing of SC, a paracentesis is created with a 15-degree side port knife at the temporal clear cornea. Then, insertion of right and left trabeculotomy probes into SC is performed to open its inner wall over 90–120°. The scleral flap and conjunctiva are sutured tightly and viscoelastic is removed from the anterior chamber. Subconjunctival injection of 4 mg dexamethasone is performed in the inferior fornix of the conjunctiva, and then antibiotic ointment is applied prior to bandage.

#### Postoperative medications

Postoperative topical medications for both surgical methods include topical dexamethasone and topical antibiotic therapies. The choice of substance and dosage is at the discretion of the surgeon. Furthermore, pilocarpine eye drops can be prescribed at the discretion of the surgeon.

In cases of transient hypotony with anterior chamber shallowing, atropine may be prescribed.

#### Emergency treatment

- Topical antiglaucomatous medication (eye drops), such as timolol, latanoprost, and dorzolamide
- Systemic antiglaucomatous medication: acetazolamide (per os/intravitreal), osmotic substances (e.g., mannitol)
- IOP-lowering procedures: angle surgery (any type of re-trabeculotomy), glaucoma drainage implants, cyclophoto-/cyclocryocoagulation, trabeculectomy

#### Post-trial care

After completing the trial, the participants will be treated and followed up according to the clinical standards which rely on the current recommendations of the medical societies for pediatric glaucoma (e.g., European Glaucoma Society, UK Pediatric Glaucoma Society). An insurance covering study-associated harms will be provided.

### Sample size

**Fig. 2:**
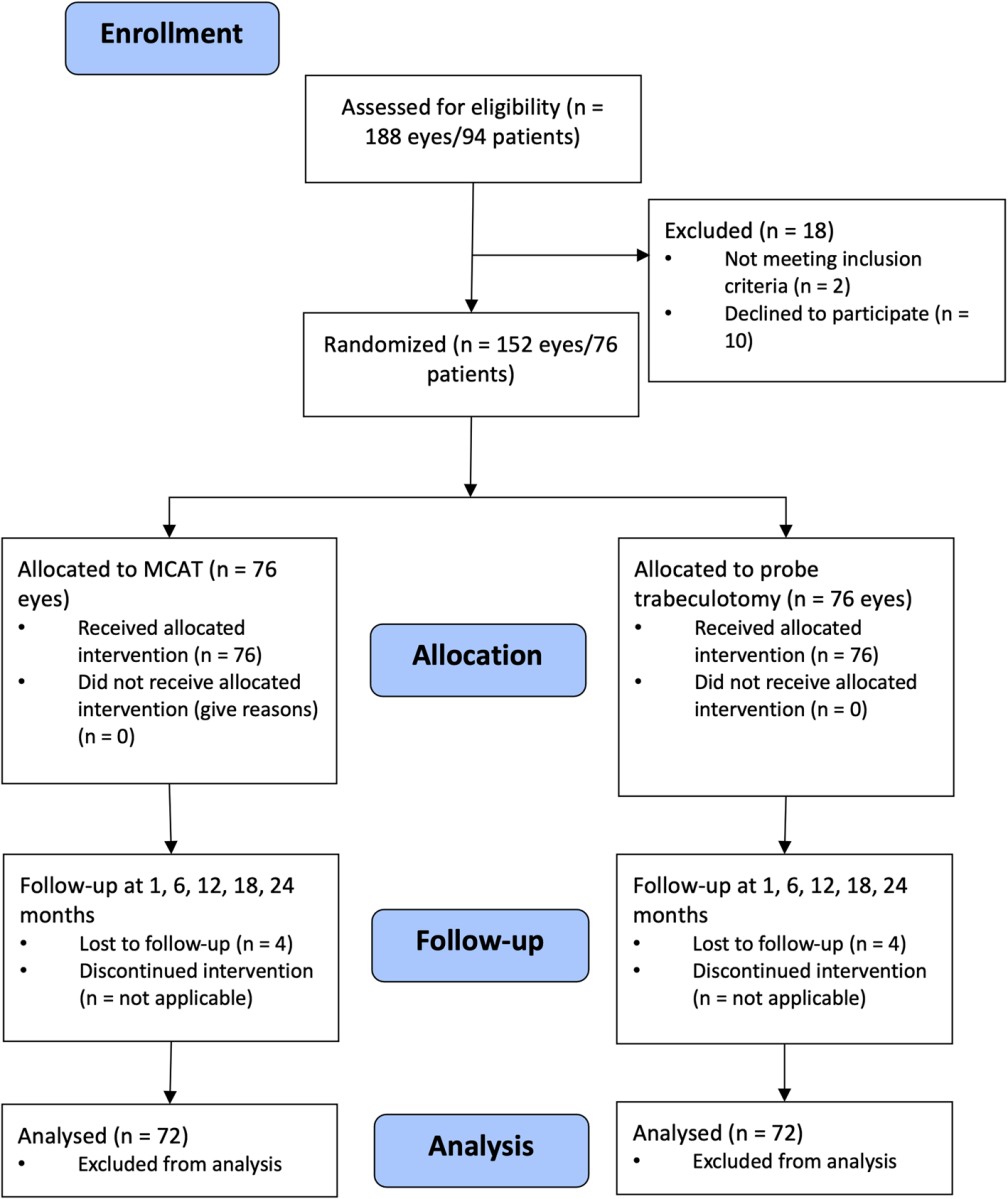
CONSORT flow diagram with expected case numbers

- El Sayed and Gawdat [8] observed 14 complete successes in 30 eyes at 2 years (47%) treated conventionally and 18 complete successes in 29 eyes treated with MCAT (62%—counting as failures those eyes in which less than 120° was possible). Therefore, we also aim to establish a difference in success rates of 15% at the 5% significance level with 80% power. In our previous experience, at most 20% of outcomes differ between both eyes of a patient. Therefore, we assume that the proportion of discordant pairs (i.e., success only with one technique in a patient) is 20%. This is equivalent to a correlation of 0.63 given overall probabilities of success of 47% and 62%.
- Power calculation has been performed using SAS PROC power. We found that with 72 patients and a correlation of 0.63 between eyes there is 80% power to detect an increase from 47% success rate to 62% success rates at the 5% level.
- We assume a dropout rate of 5% due to the empirically excellent adherence of the patients. Therefore, 76 patients are to be randomized in this study. The expected CONSORT flow diagram is presented in Fig. 2.

### Recruitment

Patients will be recruited from the childhood glaucoma consultation hours in each study center. A flyer will be provided to improve the interest in the study. A self-aid group (Bundesverband Glaukom-Selbsthilfe e.V.) has been involved in the study design and in disseminating the initiation of the study. Furthermore, recruitment of more study centers can be performed in case of low recruitment numbers.

### Interim analysis

One interim analysis of the primary endpoint is planned after 50% of the patients have completed their 6-month follow-up. As there is little previous knowledge about the correlation of outcomes, and consequently about the proportion of discordant pairs, sample size may be re-calculated after 50% of the patients have completed their 6-month follow-up.

The DSMB will evaluate the proportion of discordant pairs at 6 months. No further information on interim results shall be disclosed, thus retaining the integrity of the trial. This proportion will be taken as a proxy for the proportion of discordant pairs at 24 months, and sample size may be increased if the proportion of discordant pairs at 6 months is substantially different from 20%. The sample size may be increased up to 83 participants depending on the proportion of discordant pairs such that—given the unchanged assumption of a 15% difference—there is still 80% power to establish a 15% difference in success probability at the 5% significance level.

### Statistical methods

#### Primary outcome

The primary analysis population will be the ITT population (see definition below). The primary endpoint is complete success at 24 months. Complete success is attained if all of the following conditions are met:

- IOP < 18 mmHg at 24 months
- Successful probing > 120° in case of microcatheter-assisted trabeculotomy
- No change in surgical technique
- No topical antiglaucomatous medication in treated eye
- No systemic antiglaucomatous medication
- No revision surgery

Absolute numbers, proportions of complete success at 24 months under each intervention, and difference in proportions will be reported and displayed in cross tables. Proportions of complete success will be compared using McNemar’s test, stratified by center.

The primary estimand addresses the primary objective, i.e., to demonstrate that microcatheter-assisted trabeculotomy is superior to probe trabeculotomy regarding complete surgical success at 24 months. The population consists of all children aged ≤ 12 years with primary or secondary childhood glaucoma, requiring bilateral glaucoma surgery. The anticipated intercurrent events “topical or systemic medication,” “revision surgery,” “necessary change of surgical technique,” and, in MCAT, “less than 120° successful probing” have all been integrated into the definition of complete surgical success and are thus addressed by the *composite strategy* which considers any of these possible events also as failure.

We do not expect center effects; potential period effects will cancel out because intervention allocation is balanced across periods. However, we will check for such effects in a sensitivity analysis using a conditional logistic regression model with treatment, period, and center as covariates.

In the primary analysis, participants’ missing values of complete success at 24 months will be imputed using multiple imputation. Multiple imputation will make use of available components of the endpoint at 24 months, the corresponding variables at earlier visits, treatment, demographic variables, and possibly further variables.

#### Key secondary outcome

The analysis population will be the ITT population. The key secondary endpoint is incomplete success at 24 months. Incomplete success is attained if all of the following conditions are met:

- IOP < 18 mmHg at 24 months
- Successful probing > 120° in case of microcatheter-assisted trabeculotomy
- No change in surgical technique

Absolute numbers, proportions of incomplete success at 24 months under each intervention, and difference in proportions will be reported and displayed in cross tables. Proportions of incomplete success will be compared using McNemar’s test, stratified by center.

Here, the key secondary objective is addressed, i.e., to demonstrate that microcatheter-assisted trabeculotomy is superior to probe trabeculotomy regarding incomplete surgical success at 24 months. The corresponding estimand’s population comprises all children aged ≤ 12 years with primary or secondary childhood glaucoma, requiring bilateral glaucoma surgery. By dropping the requirements of “no topical antiglaucomatous medication in treated eye,” “no systemic antiglaucomatous medication,” and “no revision surgery,” these intercurrent events are addressed by the treatment policy strategy. “Necessary change of surgical technique” and, in MCAT, “less than 120° successful probing” still constitute surgical failure, i.e., are addressed using the composite strategy.

In the primary and key secondary analysis, participants’ missing values of incomplete success at 24 months will be imputed using multiple imputation. Multiple imputation will make use of available components of the endpoint at 24 months, the corresponding variables at earlier visits, treatment, demographic variables, and possibly further variables. Details will be specified in the statistical analysis plan.

We do not expect center effects; potential period effects will cancel out because intervention allocation is balanced across periods. However, we will check for such effects in a sensitivity analysis using a conditional logistic regression model with treatment, period, and center as covariates.

The intention-to-treat (ITT) population will include all randomized children aged ≤ 12 years who meet the inclusion criteria and are assigned to either the MCAT or PT treatment arms, regardless of whether they complete the treatment as per protocol, receive the assigned intervention, are lost to follow-up, experience protocol violations, or (not) adhere to the follow-up schedule. Children who are randomized but subsequently found to be ineligible post-randomization (e.g., misdiagnosed or found not to have childhood glaucoma) will be included in the ITT analysis unless consent is withdrawn or data is unavailable.

#### Further secondary outcomes

See Supplemental Material 1.

All analyses of secondary endpoints will be interpreted exploratory. The statistical analysis plan will be finalized before database lock.

### Data collection and management

This trial will be performed using an electronic case report form (eCRF) or remote data entry. The investigator and the trial site staff will receive system documentation, training, and support for the use of the eCRF. In case of new trial site staff, the training can be performed by personnel of the trial site. This is documented in the training log. The data are stored properly and GCP conform. Essential documents should be retained until at least 10 years after the end of the trial. Essential documents shall be archived in a way that ensures that they are readily available, upon request. The medical files of trial participants shall be retained in accordance with national legislation and in accordance with the maximum period of time permitted by the hospital, institution, or private practice.

The collected data are clinical routine data using gauged devices, and all centers use the same standards.

### Oversight and monitoring

The trial steering committee comprises two clinicians, one biostatistician, and two study coordinators who perform weekly meetings. Depending on the discussed topics, further members of the study team (clinicians, biostatisticians, monitoring, regulatory affairs, safety) will join the meeting. A trial report is provided every 2 weeks, and a periodic study newsletter summarizing amendments and the trial progress will be shared with all study centers.

Data monitoring is provided by the coordinating study center and will regularly visit the centers, review the informed consent, study data, and the investigator site files, and provide a report for the study center.

A data safety monitoring board (DSMB) consists of one glaucoma expert, one biostatistician, and one professor for history, theory, and ethics in medicine. The members are independent from the sponsor. The task of the DSMB will be to advise the principal investigator in case a change of the study design is necessary. Thus, the DSMB will be asked to evaluate the proportion of discordant pairs at 6 months to check if the sample size needs to be adapted.

### Adverse events

The participants will be carefully monitored for adverse events (AE) and serious adverse events (SAE) and their causal relationship with the interventions will be evaluated. AEs and SAEs will be documented in the eCRF. SAEs must be documented and reported to safety management of the coordinating study center within 24 h of the investigator’s awareness.

The following ophthalmological events are considered as SAE:

- Cyclo-/iridodialysis
- Choroidal hemorrhage
- Retinal detachment
- Endophthalmitis
- Phthisis

AEs and SAEs will be collected systematically and will be queried with each participant contact. The participants will be asked about both ocular and non-ocular illnesses or diseases that newly or transiently occurred during the last follow-up period, about new or transiently taken medications as well as surgical procedures since the last examination. Thus, also unanticipated AEs/SAEs will be collected. The harms will be standardized according to MedDRA for analysis and reporting. All harms will be reported in the trial publication.

### Audits

Persons (auditors) authorized by the sponsor may request access to all source documents, CRF, and other trial documentation in case of an audit. An audit program will be prepared for the study.

### Dissemination policy

The results of this trial will be published in an appropriate medical journal as well as presented at congresses. The investigators will receive the study data after publication. No further data sharing is planned.

Authorship for future trial publications will be determined in accordance with the International Committee of Medical Journal Editors (ICMJE) criteria. Individuals who have made substantial contributions to the conception or design of the study, acquisition of data, or analysis and interpretation of results, as well as those involved in drafting or critically revising manuscripts for important intellectual content, will be eligible for authorship. All contributors who meet these criteria will be appropriately acknowledged.

No professional medical writers will be employed for the preparation of any trial-related manuscripts or publications.

The statistical analysis plan will be made available upon request after the end of the trial.

## Discussion

This prospective multicenter randomized controlled study will provide reliable data on the success and safety of microcatheter-assisted 360° trabeculotomy compared to conventional probe trabeculotomy. The group sizes are adequate to detect differences in complete success after a follow-up of 24 months. The paired-eye design provides ideal comparability in a disease with highly heterogeneous phenotypes and enables to study a real-life cohort with both primary and secondary childhood glaucoma cases. It further allows a lower sample size, as interindividual systemic differences such as gender, genetics, and systemic comorbidities do not exist, which leads to a reduction of the variance. Randomization of treatment sequence minimizes selection bias. Masking of observers and patients, which has not been performed in previous randomized controlled trials comparing MCAT with probe TO [8, 10, 12], prevents observation bias. Another strength of the study will be its multicentric approach, which improves the external validity of the study results.

A limitation of the study is that unilateral glaucoma, which might be less serious and better controllable than bilateral cases, is not addressed due to the paired-eye design.

The results of this study will support the evidence-based decision-making in the treatment of childhood glaucoma, which is a rare disease with an incidence of 1:20,000 to 1:30,000 in European countries [3, 14]. It will furthermore contribute to the development of clinical guidelines.

## Trial status

Protocol version 1.2, 26.09.2024.

Date of recruitment start: 23.05.2024.

Date of recruitment completion (approximately): 23.05.2026.

## Supplementary Information

Supplementary Material 1.

Supplementary Material 2.

Supplementary Material 3.

## Data Availability

Not applicable.

## Abbreviations

AE: Adverse event
CONSORT: Consolidated Standards of Reporting Trials
DSMB: Data safety monitoring board
EUA: Examination under anesthesia
eCRF: Electronic case report form
GCP: Good Clinical Practice
IOP: Intraocular pressure
ITT: Intention to treat
MCAT: Microcatheter-assisted 360° trabeculotomy
RCT: Randomized controlled trial
SAE: Severe adverse event
SC: Schlemm’s canal
TM: Trabecular meshwork
TO: Trabeculotomy
