# Supplementary material for: Conventional probe trabeculotomy versus microcatheter-assisted 360° trabeculotomy (PIRATE) in childhood glaucoma—study protocol for a randomized controlled trial
Source: Trials. 2025 Sep 22;26:342. doi: 10.1186/s13063-025-09091-3 (PMC12455798; doi:10.1186/s13063-025-09091-3)
Supplement: Supplementary file 1 — Supplementary Material 1. [file 13063_2025_9091_MOESM1_ESM.docx]

Supplemental material 1: Further secondary outcomes

| *Outcome Domain* | *Measure* | *Metric* | *Method of aggregation* | *Time point* |
| --- | --- | --- | --- | --- |
| *Complete success* | *Success^1^* | *Value at time point* | *Absolute and relative frequency* | *1/3/6/12/18 months* |
| *Incomplete success* | *Success^2^* | *Value at time point* | *Absolute and relative frequency* | *1/3/6/12/18 months* |
| *IOP* | *Measured in mmHg* | *Difference between eyes within patient at time point* | *Mean, derived from linear regression with ΔIOP (difference between the eyes within patient) after surgery as dependent and ΔIOP at baseline as independent variable* | *1/3/6/12/18/24 months* |
| *Axial length* | *Measured in mm* | *Difference between eyes within patient at time point* | *Mean, derived from linear regression with ΔAL (difference between the eyes within patient) after surgery as dependent and ΔAL at baseline as independent variable* | *1/3/6/12/18/24 months* |
| *Corneal diameters* | *Measured in mm* | *Difference between eyes within patient at time point* | *Mean, derived from linear regression with Δcorneal diameters (difference between the eyes within patient) after surgery as dependent and Δcorneal diameters at baseline as independent variable* | *1/3/6/12/18/24 months* |
| *Visual acuity* | *LogMAR visual acuity score* | *Value at time point* | *Median* | *1/3/6/12/18/24 months* |
| *Number of amblyopia treatments* | *Count* | *Value at time point* | *Median* | *1/3/6/12/18/24 months* |
| *Number of medication drugs* | *Count* | *Value at time point* | *Median* | *1/3/6/12/18/24 months* |
| *Number of revision surgeries* | *Count* | *Value at time point* | *Median* | *1/3/6/12/18/24 months* |
| *Number of intraoperative and postoperative events* | *Count* | *Value at time point* | *Median* | *1/3/6/12/18/24 months* |

Abbreviations: ITT, Intention to treat; IOP, Intraocular pressure; AL, Axial length

^1^See definition of complete success in synopsis

^2^See definition of incomplete success in synopsis
