# Supplementary material for: Conventional probe trabeculotomy versus microcatheter-assisted 360° trabeculotomy (PIRATE) in childhood glaucoma—study protocol for a randomized controlled trial
Source: Trials. 2025 Sep 22;26:342. doi: 10.1186/s13063-025-09091-3 (PMC12455798; doi:10.1186/s13063-025-09091-3)
Supplement: Supplementary file 2 — Supplementary Material 2. [file 13063_2025_9091_MOESM2_ESM.pdf]

Director
Prof. Dr. med. Norbert Pfeiffer

study center:
Department of Ophthalmology
Mainz University Medical Center
Langenbeckstr. 1
55131 Mainz
phone: +49 (0) 6131 17-5150
email: glaukom-OP@unimedizin-mainz.de
Investigator:
Prof. Dr. med. Esther M. Hoffmann

Subject ID: _______________

Information for adolescents (12 years and older) on the research project: Surgical success rates in childhood glaucoma: Probe trabeculotomy versus microcatheter-assisted 360° trabeculotomy

Dear ,

We would like to ask you to take part in a scientific study. You will find all the important information about the study in this patient information leaflet.

Please read this information carefully. It should help you with your decision, because it is important that you understand everything.

Your parents have also received written information from us. Talk to them about the study too. Once you have read everything, you can talk about it. Your parents will certainly be able to answer most of the questions you have.

Your doctor will also talk to you about the study. You can ask anything that is unclear to you or that you still want to know. Your doctor will answer your questions.

A total of 76 patients will be included. It is planned to conduct the study at 3 centers.

This study is planned, conducted and monitored by the Department of Ophthalmology and Polyclinic of the University Medical Center Mainz.

It is supported by public funds (German Research Foundation).

The study was submitted to the responsible ethics committee. It raised no objections.

You and your parents can decide for yourself whether you want to take part in the study. Your participation is voluntary, even if your parents agree - no one is forcing you. You don't have to

decide right away. Give yourself time to think about it. If you don't want to take part in the study or withdraw later, it doesn't matter. There are no disadvantages for you. You don't have to give us a reason either.

Why is this study being conducted?

You were diagnosed with glaucoma as a child. The cause of such glaucoma is usually an abnormal development of the eye in which the outflow of water in the eye (the so-called aqueous humor) does not function normally. This causes the pressure in the eye to rise sharply. Signs of such a disease can be very different. Some children or adolescents have no symptoms, others have a foreign body sensation or pain in the eye. Sometimes the affected eye is large and red. This is due to the high pressure in the eye, which leads to damage to the optic nerve. Excessive pressure in the eye can lead to impaired vision.
Such glaucoma diseases only occur very rarely at such an early age. Because only a few children and adolescents are affected, childhood glaucoma and its treatment are not well researched. Surgery is often necessary to stop the disease. The surgeryis performed to lower your intraocular pressure. The drainage channel for the aqueous humor is restored so that your eyes can continue to see well for a long time. The drainage channel in the eye runs in a circle like a bicycle inner tube around the iris (the blue or brown part of your eye). It can either be opened completely (over 360°, see Figure 1) or only partially (over 90-120°, see Figure 2).

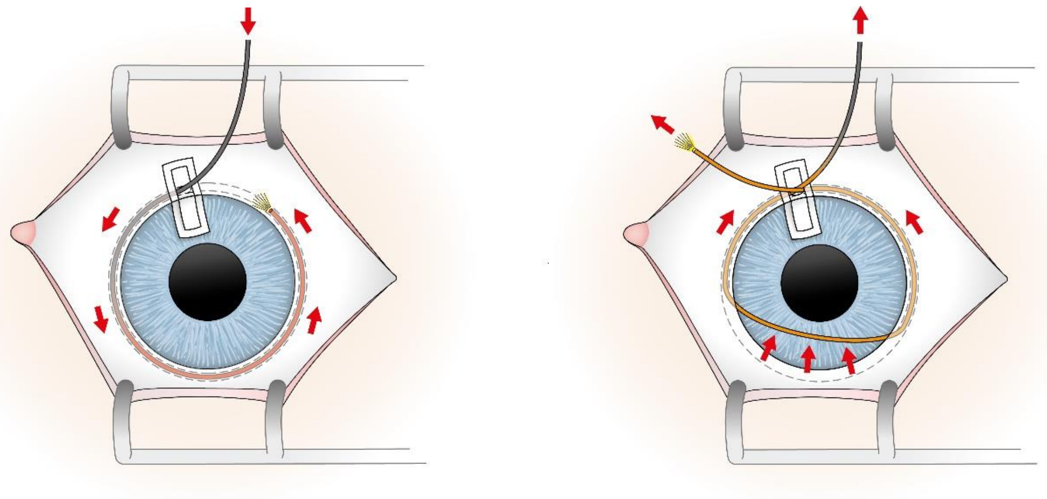

Figure 1 Complete opening using 360° trabeculotomy
Left: A thin silicone tube ("microcatheter") is threaded into the outflow channel of the eye.
Right: The ends of the silicone tube are pulled together opening the outflow channel the anterior chamber of the eye. The silicone tube then appears in the anterior chamber of the eye. The eye fluid can now drain away again.

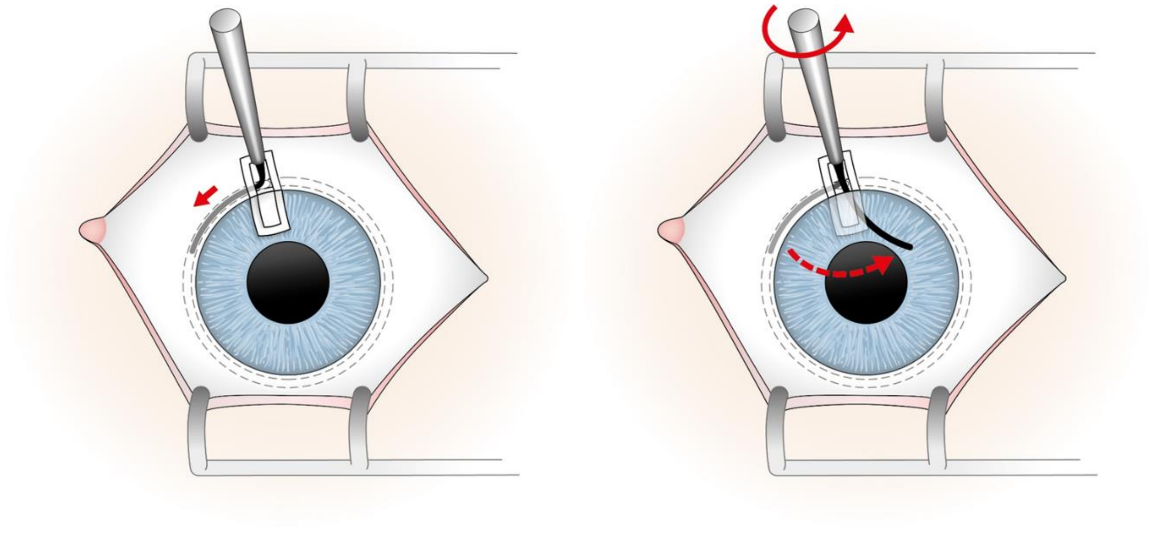

Figure 2 Partial opening using a probe trabeculotomy (90-120°)
Left: A thin metal rod ("probe") is inserted into the outflow tract.
Right: The tip of the rod is turned towards the pupil so that the outflow tract opens a short distance towards the anterior chamber of the eye. The rod then appears in the anterior chamber of the eye. The aqueous humor can now flow out again. This is done once to the right and once to the left.

The surgical methods are both called trabeculotomy. Both methods are already being used successfully in children and adolescents, but it is not yet known which method works better.

Don't worry, such an surgery is carried out in such a way that you won't notice anything and is not very painful!

With this study, we want to find out whether one of the two methods (with the silicone tube or the metal rods) works better. To do this, one of your eyes will be treated with one method and the other eye with the other method. We then want to observe which method is more successful. We will monitor your eyes in our clinic for at least 2 years. The follow-up checks

https://etic.med.tum.de

would also take place if you did not take part in the study. Therefore, there is no great additional expense for you.

Our clinical experience to date with both surgical techniques has shown the following possible advantages and disadvantages of each method:

The advantage of the probe trabeculotomy is the slightly shorter (approx. 2-3 minutes) surgery time and the slightly less bleeding from the blood-filled Schlemm's canal, as only 120° is opened.

The advantage of the 360° trabeculotomy is the circular opening of the canal and the assumption of greater pressure reduction. The disadvantage may be increased bleeding (hyphaema) and therefore possibly faster scarring than with the conventional method.

As these assumptions have not yet been scientifically confirmed, we hope that this study will enable us to better assess the advantages and disadvantages.

How does the study work?

The study is expected to last 2 years for each participant.

We will try to treat both eyes at the same time so that you have to spend as little time as possible in hospital. However, sometimes it is also better to leave a little space between the surgerys on the two eyes. We will discuss this with you and your parents.
On the day after the surgery, there will be a short examination, a so-called ward round, which will take place on the ward where you will sleep before and after the surgery. As a rule, you will be able to go home again in the following days. After that, 6 more check-ups are planned, spread over the following 2 years: After 4 weeks, 3 months, 6 months, 12 months, 18 months and 24 months. These check-ups take about 2 hours. As already mentioned, such examinations usually also take place if the surgery is not part of a study. This means that you will not be examined more often than usual. A regular follow-up check is simply important to ensure that the intraocular pressure is within a good range!

In most cases, both eyes are affected by the disease. In the study, one of your eyes is completely opened during the surgery and the other is partially opened. This allows you to compare the success of both methods. Both surgical procedures are regularly performed on children with glaucoma like yours. They are therefore already tried and tested in children. If you take part in this study, a lot will be drawn to determine which eye will be operated on with which method. You will not notice the difference. Even the doctor and your parents will not know which eye was operated on and how. Only the surgeon knows this.

https://etic.med.tum.de

Can it be unpleasant or can something bad happen?

If you feel anything strange after the surgery, tell your parents or doctor immediately. This is very important so that we can help you properly. It is common to have a small amount of bleeding in the eye after such an surgery, which is not serious. It is possible that such bleeding may result in poor vision for a few days. However, this usually resolves quickly.

The doctors will inform you and your parents separately about the medical risks of the surgery. Participation in the study is not associated with any additional medical risks. Your doctor will check your eyes regularly to monitor the healing process.

Are there any personal benefits from participating in the study?

You probably won't benefit from taking part in the study yourself. However, the results of the study may help other people in the future.

What are the risks associated with participating in the study?

Participation in the study is not associated with any additional medical risks.

What other options are there outside the study?

In addition to the trabeculotomy, there are other surgical procedures. These are normally only used if the trabeculotomy does not work well enough.

Are there any additional costs?

There are no additional costs for your parents, for you or for the health insurance company as a result of your participation in the study.

Was insurance taken out for this study?

During the study, the usual insurance cover of the Mainz University Medical Center applies. Insurance cover is provided if the doctor or another employee of the trial site is accused of culpable misconduct.

In this clinical study, all study participants are also insured under a volunteer and commuting accident insurance policy. The scope of the insurance cover can be found in the insurance documents that you will receive.

If you suspect that your child's health has been damaged or existing conditions have been exacerbated by participation in the clinical trial, you must inform the insurer immediately.

Name and address of the insurance company: HDI Global SE
Proactiv Platz 1
40721 Hilden
phone: +49 211 7482-0
email: Christoph.Schmidt@hdi.global

Annual contract for volunteer and commuting accident insurance no: 76 307104 03017/03059

directly, if necessary with the support of your study doctor, so as not to jeopardize your child's insurance cover. If your study doctor supports you in this, you will receive a copy of the notification. If you notify the insurer directly, please also inform your study doctor.

You must cooperate in clarifying the cause or extent of the damage and do everything possible to avert and minimize the damage.

A copy of the insurance confirmation and the insurance conditions will also be provided.

Information on data protection

In this study, the University Medical Center of Johannes Gutenberg University Mainz, represented by the Executive Board, is responsible for data processing. The legal basis for the processing is personal consent (Art. 6 para. 1 lit. a, Art. 9 para. 2 lit. a GDPR).

The data will be treated confidentially at all times.

The data is collected exclusively for the purpose of this study described above and is only used within this framework.

The data collected also includes personal identifying data such as name, address, date of birth and sensitive personal health data.

For the study, we would also like to record which country you or your parents come from.

All data that can be used to find out that it is you who is taking part in the study, e.g. your name or date of birth, will be replaced by a code (pseudonymized). Only we know the code. This makes it almost impossible for others to find out that it is you.

The data is stored at the Interdisciplinary Center for Clinical Studies, University Medical Center Mainz.

We only store the personal data for as long as is necessary for the above-mentioned purpose. The data will be deleted at the latest 10 years after discontinuation or termination of the study.

Consent to the processing of your data is voluntary. You can revoke it together with your parents at any time. You do not have to justify this and you will not suffer any disadvantages as a result.

If you would like to know more about what happens to your data, you can take a look at the information we have given your parents.

Who else can you ask questions to?

If you have any further questions, please feel free to contact us. We will be happy to answer all your questions.

email: glaukom-op@unimedizin-mainz.de

phone: 06131-17 5150

https://etic.med.tum.de

Director
Prof. Dr. med. Norbert Pfeiffer

study center:
Department of Ophthalmology
Mainz University Medical Center
Langenbeckstr. 1
55131 Mainz
phone: +49 (0) 6131 17-5150
email: glaukom-OP@unimedizin-mainz.de
Investigator:
Prof. Dr. med. Esther M. Hoffmann

Subject ID: _______________

Information for parents/guardians on the research project: Surgical success rates in childhood glaucoma: Probe trabeculotomy versus microcatheter-assisted 360° trabeculotomy

Dear parents,

Dear guardians,

We would like to ask you to allow your child to take part in a scientific study. In this information you will find everything you need to know about the study.

Please read this information carefully. Your doctor will talk to you about the study and answer your questions.

A total of 76 patients will be included. It is planned to conduct the study at 3 centers (Mainz, Cologne, Leuven/Belgium).

This study is planned, conducted and monitored by the Department of the University Medical Center Mainz. It is funded by the German Research Foundation.

The study was submitted to the responsible ethics committee. It raised no objections.

Your decision to allow your child to participate in the study is voluntary. You can also terminate your child's participation at any time during the course of the study (verbally, in writing or in text form). You do not have to give reasons for this. This will not result in any disadvantages for you or your child. In particular, your decision will not result in any disadvantages for your child's medical treatment or the relationship with the doctor treating him/her.

If you have any further questions about the study now or later, please feel free to contact us. Please contact Prof. Dr. Esther M. Hoffmann (email: glaukom-op@unimedizin-mainz.de, telephone: +49 (0) 6131/17 5150).

Why is this study being conducted?

Your daughter/son is suffering from childhood glaucoma. The cause of such glaucoma is usually incomplete development of the eye, in which the outflow of water in the eye (the so-called aqueous humor) does not function normally. As a result, the intraocular pressure rises sharply and the optic nerve is damaged. This is usually accompanied by clouding of the cornea and growth of the eyeball. Affected children often suffer from sensitivity to light, tearing, eyelid spasms and rubbing their eyes.
Glaucoma only occurs very rarely at such an early age. Because only a few children are affected, childhood glaucoma and its treatment are not well researched.
Treatment usually consists of an surgery to prevent blindness. The surgery of first choice is trabeculotomy, in which the outflow pathway of the aqueous humor is restored. The collecting duct that surrounds the anterior chamber of the eye and into which the aqueous humor normally drains is exposed and opened to the anterior chamber of the eye. There are 2 different surgical methods for this: with the help of an illuminated silicone tube, the canal can be opened completely over 360° (360° trabeculotomy, see Figure 1). Metal probes can also be used to open the canal by 90 to 120° (probe trabeculotomy, see Figure 2).

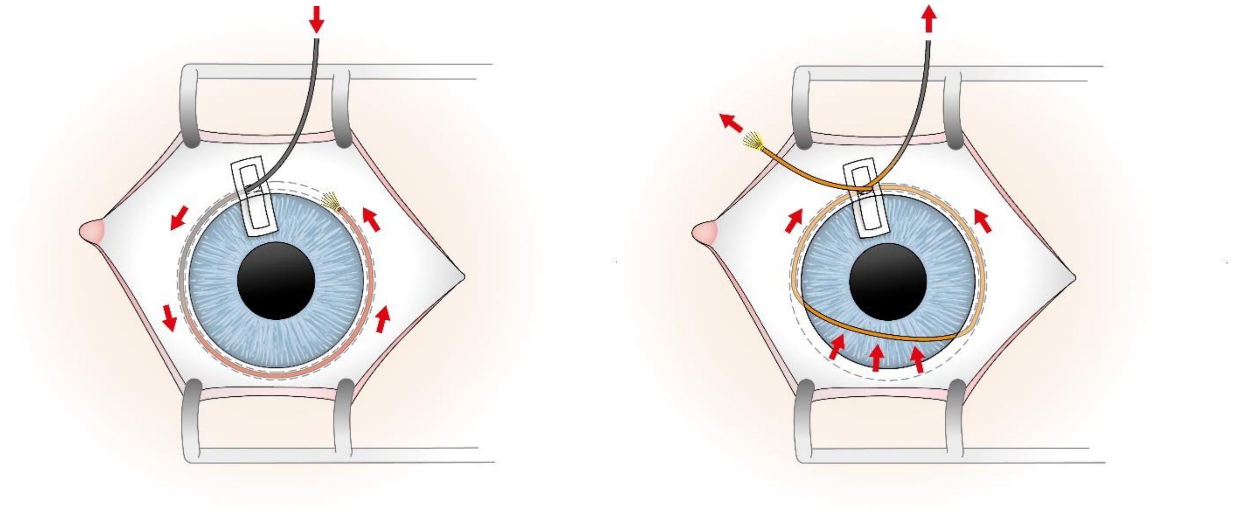

Figure 1 Complete opening using 360° trabeculotomy
Left: A thin silicone tube ("microcatheter") is threaded into the outflow channel of the eye.
Right: The ends of the silicone tube are pulled together so that the outflow channel opens towards the anterior chamber of the eye. The silicone tube then appears in the anterior chamber of the eye. The eye fluid can now drain away again.

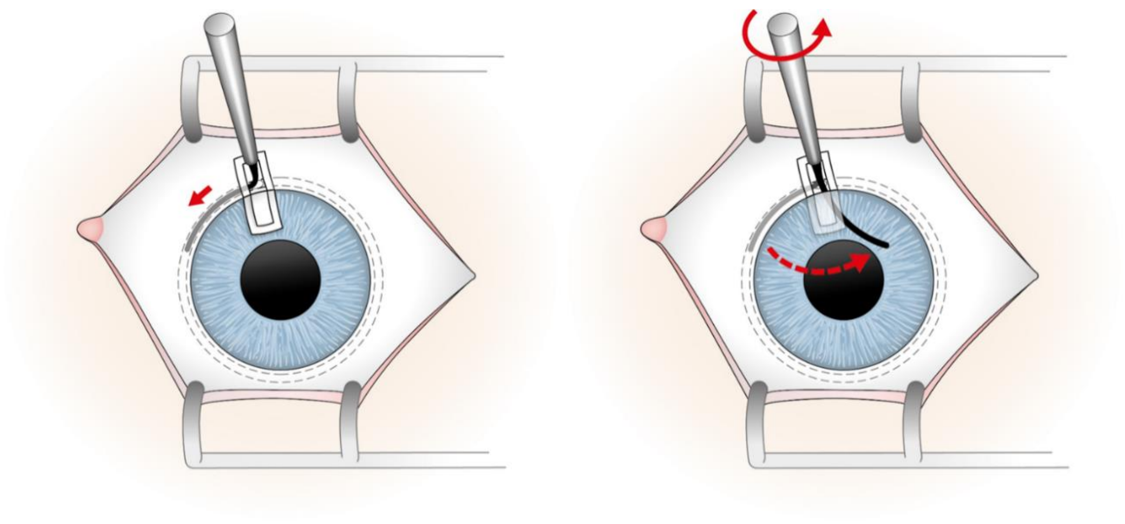

Figure 2 Partial opening by means of probe trabeculotomy (90-120°)
Left: A thin metal rod ("probe") is inserted into the outflow tract.
Right: The tip of the probe is turned towards the pupil so that the outflow tract opens a short distance towards the anterior chamber of the eye. The probe then appears in the anterior chamber of the eye. The aqueous humor can now flow out again. This is carried out in both the right and left direction.

Our clinical experience to date with both surgical techniques has shown the following possible advantages and disadvantages of each method:

The advantage of the probe trabeculotomy is the slightly shorter (approx. 2-3 minutes) surgery time and the slightly less bleeding from the blood-filled Schlemm's canal, as only 120° is opened.

The advantage of the 360° trabeculotomy is the circular opening of the canal and the assumption of greater pressure reduction. The disadvantage is the increased bleeding (hyphaema) and therefore possibly faster scarring than with the conventional method.

As these assumptions have not yet been scientifically confirmed, we hope that this study will enable us to better assess the advantages and disadvantages.

In this study, we want to find out which type of trabeculotomy is more successful. To this end, intraocular pressure will be compared 2 years after surgery. One eye will receive a 360° trabeculotomy, the other a probe trabeculotomy. This will ensure optimal comparability between the two surgical procedures.

How does the study work?

The study is expected to last 2 years for each participant.

https://etic.med.tum.de

If possible, both eyes are operated on in one session. The aim of this procedure is to save on anesthesia and to allow both eyes to be relieved at the same time. If this is not possible, the second eye can also be operated on at a later date.
On the first day after the surgery, a visit is made as part of the inpatient stay. Otherwise, 6 follow-up examinations are planned at the clinic. They take place after 4 weeks, 3 months, 6 months, 12 months, 18 months and 24 months. Normally, awake examinations are sufficient; however, an anesthetic examination may be necessary if the examination conditions are poor or if it is suspected that the intraocular pressure has not been reduced sufficiently. Two hours should be allowed for an examination on an awake child. For an examination under anesthesia, 2 days should be planned and an inpatient stay is necessary. The follow-up examinations correspond to the normal rhythm and the normal examination spectrum of the clinical routine, so that no additional examinations and appointments will be necessary. All examinations would be carried out in the same way if your child had a trabeculotomy outside of the study.

As each child receives both surgical methods that are compared in the study, it is determined at random (like tossing a coin) which eye receives which surgery (randomization). Randomization ensures that the study results are not falsified by unknown influencing factors, thus ensuring comparability of the surgical methods.

Neither you nor your doctor (who will examine your child) will know which eye has had which surgery before the study is completed. This is called blinding and is necessary to prevent any influence on the results. However, in an emergency, your doctor can always find out which eye has had which surgery. Only the surgeon knows this.

Are there any personal benefits from participating in the study?

There is no personal benefit to be expected from participating in the study. However, the results of the study may help other people in the future.

What are the risks associated with participating in the study?

Participation in the study is not associated with any additional medical risks. You and your child will be informed separately about the risks of the surgery.

What other options are there outside the study?

In clinical routine, both the 360° trabeculotomy and the probe trabeculotomy are performed. We are trying to find out which surgical method works better with this study.
Other surgical methods are the insertion of a drainage implant or the kryotherapy or

https://etic.med.tum.de

photocoagulation of the ciliary body. However, these surgical methods are the second choice compared to trabeculotomy.

Are there any additional costs?

Neither you nor your health insurance company will incur any additional costs by participating in the study.

Was insurance taken out for this study?

During the study, the usual insurance cover of the Mainz University Medical Center applies. Insurance cover is provided if the doctor or another employee of the trial site is accused of culpable misconduct.

In this clinical study, all study participants are also insured under a volunteer and commuting accident insurance policy. The scope of the insurance cover can be found in the insurance documents that you will receive.

If you suspect that your child's health has been damaged or existing conditions have been exacerbated by participation in the clinical trial, you must inform the insurer immediately.

Name and address of the insurance company: HDI Global SE
Proactiv-Platz 1
40721 Hilden
phone: +49 (0) 211 7482-0
email: Christoph.Schmidt@hdi.global

Annual contract for volunteer and commuting accident insurance no: 76 307104 03017/03059

directly, if necessary with the support of your study doctor, so as not to jeopardize your child's insurance cover. If your study doctor supports you in this, you will receive a copy of the notification. If you notify the insurer directly, please also inform your study doctor.

You must cooperate in clarifying the cause or extent of the damage and do everything possible to avert and minimize the damage.

A copy of the insurance confirmation and the insurance conditions will also be provided.

https://etic.med.tum.de

Information on data protection

In this study, the University Medical Center of Johannes Gutenberg University Mainz, represented by the Executive Board (Langenbeckstr. 1, 55131 Mainz, phone +49 (0) 6131/17-0, website: http://www.unimedizin-mainz.de/) is responsible for data processing. The legal basis for the processing is personal consent (Art. 6 para. 1 lit. A, Art. 9 para. 2 lit. A GDPR). The data will be treated confidentially at all times.

The data is collected exclusively for the purpose of this study described above and is only used within this framework.

The data collected also includes personal identifying data such as name, address, date of birth and sensitive personal health data.

The data that we process from your child is personal data in accordance with Art. 4 No. 1 -GDPR or special categories of personal data in the form of health data in accordance with Art. 4 No. 15 -GDPR-.

Specifically, we process the following data: Data collected as part of your child's medical history such as family history of glaucoma, date of birth and onset of disease etc.) or measured (highest intraocular pressure ever measured, current intraocular pressure, visual acuity, eye length, revision surgery and other ocular parameters).

We also collect other sensitive personal data. This includes your ethnic origin (country) and the degree to which you are related to each other.

All data that could be used to directly identify your child, e.g. their name or date of birth, is replaced by an identification code (pseudonymized). This makes it almost impossible for unauthorized persons to identify your child. Identification can only take place via the pseudonymization list. This is only accessible to the scientists involved.

The data is stored at the Interdisciplinary Center for Clinical Studies at the Mainz University Medical Center.

We only store the personal data for as long as is necessary for the above-mentioned purpose. The data will be deleted at the latest 10 years after discontinuation or termination of the study, unless statutory retention periods prevent this.

We do not transfer personal data to other institutions in Germany, the EU, to a third country outside the EU or to an international organization.

The data is passed on to the following institutions that are not directly involved in the treatment:

https://etic.med.tum.de

- The IZKS (Interdisciplinary Center for Clinical Studies) of the University Medical Center Mainz is creating a study database on behalf of the Eye Clinic in which the pseudonymized data of your child will be stored.
- The IMBEI (Institute for Medical Biometry, Epidemiology and Informatics) of the Mainz University Medical Center will analyze the pseudonymized data.

The data is published in anonymized form.

We do not transfer personal data to other institutions in Germany, the EU, to a third country outside the EU or to an international organization.

Consent to the processing of your and your child's data is voluntary. You can withdraw your consent at any time without giving reasons and without any disadvantages for you or your child. No more data will then be collected. This does not affect the lawfulness of the processing carried out on the basis of the consent until revocation.

In the event of revocation, you can request the deletion of the data collected. The data can also be used in anonymized form if you consent to this at the time of your revocation.

You have the right to receive information about the data, also in the form of a copy free of charge. In addition, you may request the rectification, blocking, restriction of processing or erasure and, where applicable, the portability of the data. You also have the right to object to the processing.

The personal data collected is not subject to decisions based solely on automated processing (e.g. profiling).

In these cases, please contact us if you have any further questions about data protection and the handling of data or in the event of revocation:

Prof. Dr. med. Esther M. Hoffmann
Department of Ophthalmology
University Medical Center Mainz
Langenbeckstraße 1
55131 Mainz
telephone: +49 (0) 6131/17 5150
email: ehoffman@uni-mainz.de

If you have any questions about data processing and compliance with data protection, please contact the Data Protection Officer of the Mainz University Medical Center:

Langenbeckstraße 1
55131 Mainz
phone: +49 (0)6131/17-0

https://etic.med.tum.de

email: datenschutz@unimedizin-mainz.de

You also have the right to lodge a complaint with any data protection supervisory authority. You can find a list of the supervisory authorities in Germany at: https://www.bfdi.bund.de/DE/Infothek/Anschriften_Links/anschriften_links-node.html

You can contact the supervisory authority responsible for the Mainz University Medical Center, the State Commissioner for Data Protection and Freedom of Information, at

P.O. Box 30 40, 55020 Mainz
Hintere Bleiche 34, 55116 Mainz
phone: +49 (0) 6131 8920-0
fax: +49 (0) 6131 8920-299
email: poststelle@datenschutz.rlp.de
https://www.datenschutz.rlp.de

https://etic.med.tum.de

Director
Prof. Dr. med. Norbert Pfeiffer

study center:
Department of Ophthalmology
Mainz University Medical Center
Langenbeckstr. 1
55131 Mainz
phone: +49 (0) 6131 17-5150
email: glaukom-OP@unimedizin-mainz.de
Investigator:
Prof. Dr. med. Esther M. Hoffmann

Subject ID: _______________

Informed consent for the research project: Surgical success rates in pediatric glaucoma: Probe trabeculotomy versus microcatheter-assisted 360° trabeculotomy

I have been informed about the study by ____________________________. I have received and read the written information and declaration of consent for the above-mentioned study. I have been informed in detail in writing and verbally about the purpose and course of the study, the opportunities and risks of participation and the associated rights and obligations. I was also -comprehensively informed about the processing of my personal data in accordance with Art. 13 -GDPR. I had the opportunity to ask questions. These were answered satisfactorily and in full. In addition to the written information, the following points were discussed

____

____

____

My consent for my child

_______________________________________, born on _______________________,

participation in the study is voluntary. I have the right to withdraw this consent at any time without giving reasons and without any disadvantages for me or my child.

The processing and use of personal data for the above-mentioned study is carried out exclusively as described in the information on the study.

I have understood and agree to this,

1. that my personal data required for the purpose of the above-mentioned study (in particular health data and information on my child's ethnic origin) will be collected by the study doctor and recorded and processed in pseudonymized form, including on electronic data carriers;

2. that the study results will be published in an anonymous form that does not allow any conclusions to be drawn about my person;

3. that my data will be processed exclusively for the above-mentioned purposes and only by study staff or the named recipients or categories of recipients

We have received one copy of the information and consent. One copy remains at the test center.

Consent of the patient:

Patient: I give my consent to participate in the above-mentioned study.

____
Name of the patient

____
Place, date Signature **of the patient**

Consent of the legal guardian(s):

I hereby consent to my child taking part in the above-mentioned study.

____
Name of the **first legal guardian** in block capitals

____
Place, date Signature of the **first legal guardian**

____
Name of the **second legal guardian** in block capitals

____
Place, date Signature of the **second legal guardian**

If only one parent or legal guardian signs, the signature confirms that this is done in agreement with the other parent or that the person signing has sole custody:
Please comment accordingly by the sole custodian:

____
Name of the sole custodian in block capitals

____
Place, date Signature of the **sole custodian**

Witness/Interpreter

I was present throughout the process of informing the participant and confirm that the information about the aims and procedures of the study was adequately communicated, that the participant (or their legal representative) clearly understood the study and that consent to participate in the study was given voluntarily.

____
Name and qualification of the witness/interpreter in block capitals

____
Place, date Signature of the **witness/interpreter**

Informing physician:

I have conducted the informed consent discussion and obtained the consent of the patient's legal guardians and the patient.

I hereby confirm that I have informed the above-mentioned legal guardians and the patient about the nature, purpose and foreseeable effects of the study. All questions have been answered and I have received a copy of the study information and informed consent form. The legal guardians and the patient have agreed to voluntary participation in the study with their signature.

____
Name of the informing investigator in block capitals

____
Place, date Signature of the **informing investigator**

UNIVERSITÄTSmedizin. MAINZ
Augenklinik und Poliklinik

Director
Prof. Dr. med. Norbert Pfeiffer

Study center:
Department of Ophthtalmology
Mainz University Medical Center
Langenbeckstr. 1
55131 Mainz
phone: +49 (0) 6131 17-5150
email: glaukom-OP@unimedizin-mainz.de
Investigator:
Prof. Dr. med. Esther M. Hoffmann

Subject ID: _______________

Information for children (7-11 years): Surgical success rates in childhood glaucoma: Probe trabeculotomy versus microcatheter-based 360° trabeculotomy

Dear ,

you are being treated in our clinic for your glaucoma. Signs of such a disease can be very different. Some children have no symptoms, others have a foreign body sensation or pain in their eyes. Sometimes the affected eye is large and red. This is due to the high pressure in the eye, which also often restricts vision. In order to get well, you will need a surgery.

Children like you are currently being treated with various types of surgery. However, because the disease is so rare, no research has yet been carried out into which surgical method is better. We doctors now want to figure this out. We would like to know whether you would like to take part in this research project, in which other children are also participating.

You can decide together with your parents whether you would like to take part in the research project. No one is forcing you to take part. You can also simply say no. You don't have to decide straight away. Give yourself time to think about it. Your parents will certainly be able to answer most of the questions you have. Otherwise, you can also talk to the doctors about this research project and ask questions.

What is done in the research project and what happens to you?

The surgery is performed to lower your intraocular pressure. This is necessary because too much pressure in the eye can lead to poor vision. To ensure that your eyes can continue to see

https://etic.med.tum.de

well for a long time, the drainage channel for the aqueous humor is unblocked. The drainage channel in the eye runs in a circle like a bicycle inner tube around the iris (the blue or brown part of your eye). It can either be opened completely (over 360°, see Figure 1) or only partially (over 90-120°, see Figure 2) so that the aqueous humor can drain away again.

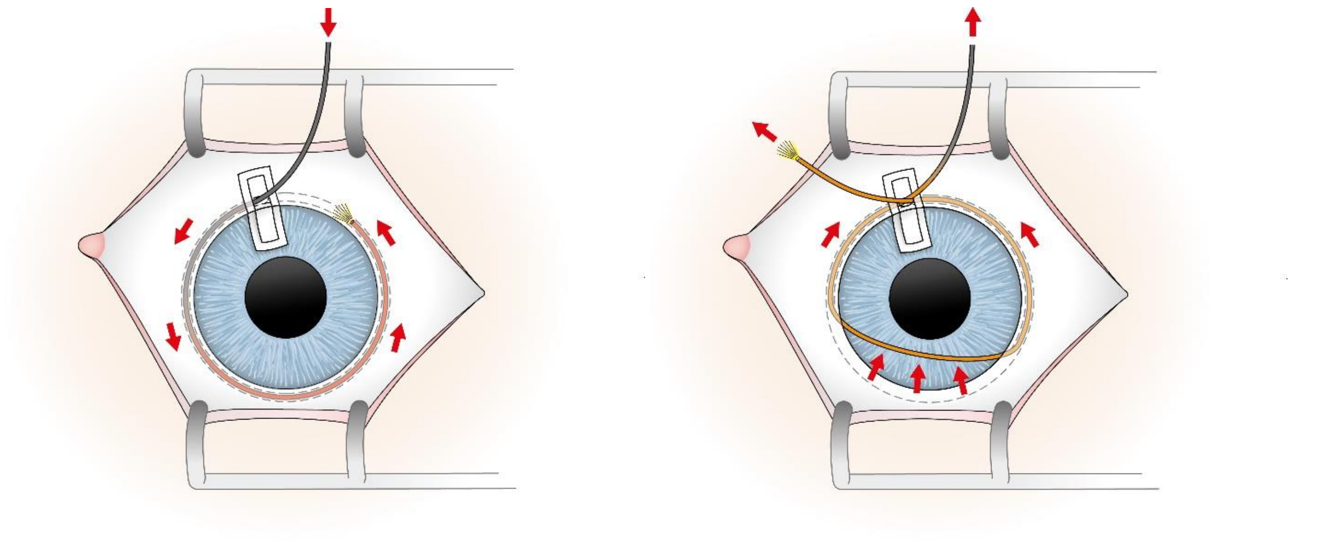

Figure 1 Complete opening
Left picture: A thin rubber tube is threaded into the drainage channel of the eye.
Right picture: The ends of the rubber tube are pulled together. The drainage canal opens all around and the aqueous humor can drain out again.

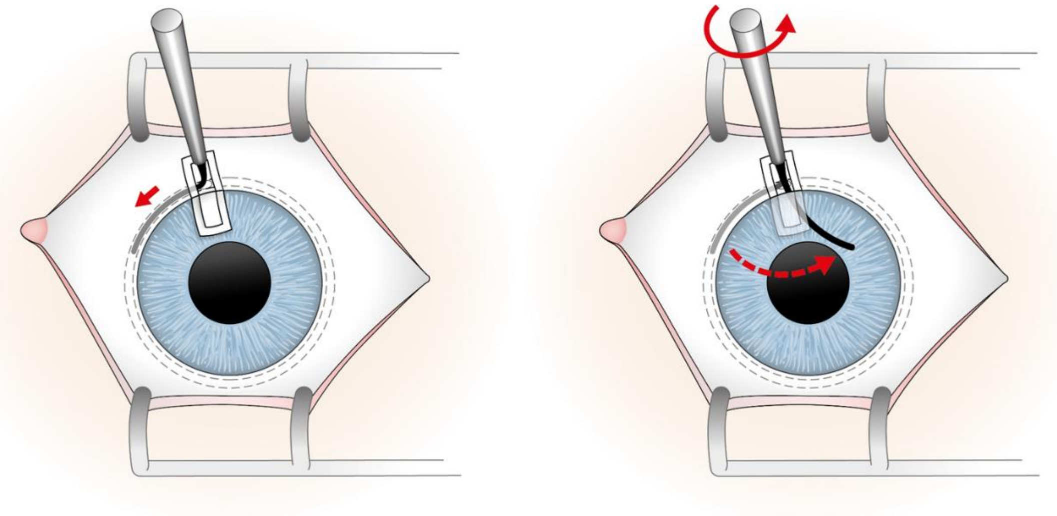

Figure 2 Partial opening
Left picture: A thin metal rod is inserted into the outflow canal.
Right picture: The tip of the rod is turned towards the pupil, once to the right and once to the left. The drainage canal opens in the upper part of the pupil and the aqueous humor can drain out again.

https://etic.med.tum.de

You don't have to worry, you won't be able to see that the drainage channel is open later.

We carry out various eye examinations before the surgery, some of which are repeated after the surgery. Further check-ups are also planned, 6 in total. These will take place after 4 weeks, 3 months, 6 months, 12 months, 18 months and 24 months. The examinations would also take place if you are not taking part in the research project. This means that you will not be examined more often than usual.

In most cases, both eyes are affected by the disease. In the research project, one of your eyes is completely opened during the surgery and the other is partially opened. This allows the success of both methods to be compared. Both surgical procedures are regularly performed on children with glaucoma like yours. They are therefore already tried and tested in children. If you take part in this research project, a lot will be drawn to determine which eye will be operated on with which method. You won't notice the difference. Even the doctor who examines you and your parents will not know which eye was operated on and how. Only the surgeon knows this.

Can it be unpleasant or can something bad happen?

If you feel anything strange after the surgery, tell your parents or doctor immediately. This is very important so that we can help you properly. It is common to have a small amount of bleeding in the eye after such a surgery, which is not serious. It is possible that such bleeding may result in poor vision for a few days. However, this usually goes away quickly.

You and your parents will be informed separately by the doctors about the medical risks of the surgery. Participation in the research project is not associated with any additional medical risks. Your doctor will check your eyes regularly to monitor the healing process.

Do you have to participate until the end of the research project?

Because you are taking part in the research project voluntarily, you can stop at any time. You don't have to give us a reason. If you stop, you will continue to be treated as if you had not taken part in the research project.

What happens to your data?

The data collected will be stored on paper and electronically. All data from this research project will be deleted after 10 years at the latest.

Who else can you ask questions to?

If you have any further questions, please feel free to contact us. We will be happy to answer all your questions.

https://etic.med.tum.de

email: glaukom-op@unimedizin-mainz.de

phone: 06131-17 5150

https://etic.med.tum.de

JGU UNIVERSITÄTSmedizin.
Augenklinik und Poliklinik MAINZ

Director
Prof. Dr. med. Norbert Pfeiffer

Study center:
Department of Ophthtalmology
Mainz University Medical Center
Langenbeckstr. 1
55131 Mainz
phone: +49 (0) 6131 17-5150
email: glaukom-OP@unimedizin-mainz.de
Investigator:
Prof. Dr. med. Esther M. Hoffmann

Subject ID: _______________

Information for parents/guardians on the research project: Surgical success rates in childhood glaucoma: Probe trabeculotomy versus microcatheter-assisted 360° trabeculotomy

Dear parents,

Dear guardians,

We would like to ask you to allow your child to take part in a scientific study. In this information you will find everything you need to know about the study.

Please read this information carefully. Your doctor will talk to you about the study and answer your questions.

A total of 76 patients will be included. It is planned to conduct the study at 3 centers (Mainz, Cologne, Leuven/Belgium).

This study is planned, conducted and monitored by the Department of Ophthalmology of the University Medical Center Mainz. It is funded by the German Research Foundation.

The study was submitted to the responsible ethics committee. It raised no objections.

Your decision to allow your child to participate in the study is voluntary. You can also terminate your child's participation at any time during the course of the study (verbally, in writing or in text form). You do not have to give reasons for this. This will not result in any disadvantages for you or your child. In particular, your decision will not result in any disadvantages for your child's medical treatment or the relationship with the doctor treating him/her.

https://etic.med.tum.de

If you have any further questions about the study now or later, please feel free to contact us. Please contact Prof. Dr. Esther M. Hoffmann (email: glaukom-op@unimedizin-mainz.de, telephone: +49 (0) 6131/17 5150).

Why is this study being conducted?

Your daughter/son is suffering from childhood glaucoma. The cause of such glaucoma is usually incomplete development of the eye, in which the outflow of water in the eye (the so-called aqueous humor) does not function normally. This causes the intraocular pressure to rise sharply, resulting in damage to the optic nerve. This is usually accompanied by clouding of the cornea and growth of the eyeball. Affected children often suffer from sensitivity to light, tearing, eyelid spasms and rubbing their eyes.
Glaucoma only occurs very rarely at such an early age. Because only a few children are affected, childhood glaucoma and its treatment are not well researched.
Treatment usually requires a surgery to prevent blindness. The surgery of first choice is trabeculotomy, in which the outflow pathway of the aqueous humor is restored. The collecting duct that surrounds the anterior chamber of the eye and into which the aqueous humor normally drains is exposed and opened to the anterior chamber of the eye. There are 2 different surgical methods for this: with the help of an illuminated silicone tube, the canal can be opened completely over 360° (360° trabeculotomy, see Figure 1). Metal probes can also be used to open the canal by 90 to 120° (probe trabeculotomy, see Figure 2).

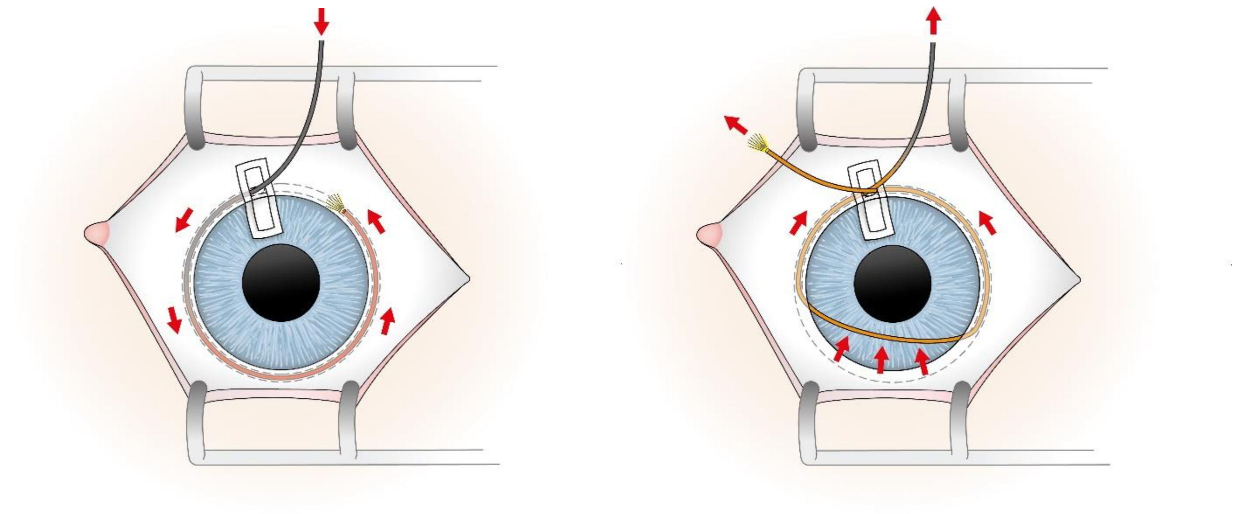

Figure 1 Complete opening using 360° trabeculotomy
Left: A thin silicone tube ("microcatheter") is threaded into the outflow channel of the eye.
Right: The ends of the silicone tube are pulled together so that the outflow channel opens towards the anterior chamber of the eye. The silicone tube then appears in the anterior chamber of the eye. The eye fluid can now drain away again.

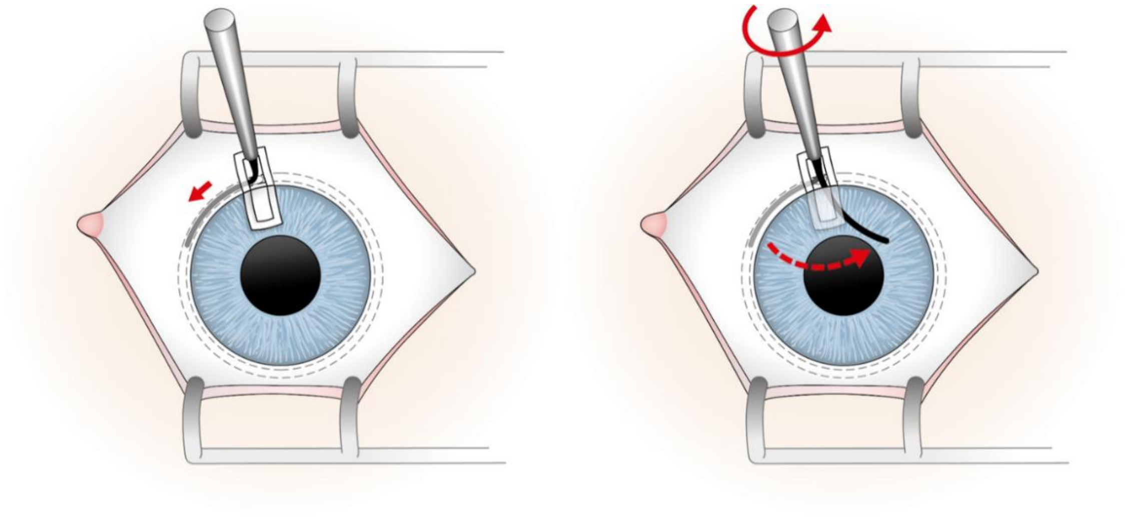

Figure 2 Partial opening by means of probe trabeculotomy (90-120°)
Left: A thin metal rod ("probe") is inserted into the outflow tract.
Right: The tip of the probe is turned towards the pupil so that the outflow tract opens a short distance towards the anterior chamber of the eye. The probe then appears in the anterior chamber of the eye. The aqueous humor can now flow out again. This is carried out in both the right and left direction.

Our clinical experience to date with both surgical techniques has shown the following possible advantages and disadvantages of each method:

The advantage of the probe trabeculotomy is the slightly shorter (approx. 2-3 minutes) surgery time and the slightly less bleeding from the blood-filled Schlemm's canal, as only 120° is opened.

The advantage of the 360° trabeculotomy is the circular opening of the canal and the assumption of greater pressure reduction. The disadvantage is the increased bleeding (hyphaema) and therefore possibly faster scarring than with the conventional method.

As these assumptions have not yet been scientifically confirmed, we hope that this study will enable us to better assess the advantages and disadvantages.

In this study we would like to find out which type of trabeculotomy is more successful. For this purpose, the intraocular pressure 2 years after surgery will be compared. One eye will receive a 360° trabeculotomy, the other a probe trabeculotomy. This will ensure optimal comparability between the two surgical procedures.

How does the study work?

The study is expected to last 2 years for each participant.

https://etic.med.tum.de

If possible, both eyes are treated on in one session. The aim of this procedure is to save on anesthesia and allow both eyes to be relieved at the same time. If this is not possible, the second eye can also be treated on at a later date.

On the first day after the surgery, a visit is made as part of the inpatient stay. Otherwise, 6 follow-up examinations are planned at the clinic. They take place after 4 weeks, 3 months, 6 months, 12 months, 18 months and 24 months. Normally, awake examinations are sufficient; however, an anesthetic examination may be necessary if the examination conditions are poor and if it is suspected that the intraocular pressure has not been reduced sufficiently. Two hours should be allowed for an examination on an awake child. For an examination under anesthesia, 2 days should be planned and an inpatient stay is necessary. The follow-up examinations correspond to the normal rhythm and the normal examination spectrum of the clinical routine, so that no additional examinations and appointments will be necessary. All examinations would be carried out in the same way if your child had a trabeculotomy outside of the study.

As each child receives both surgical methods that are compared in the study, it is determined at random (like tossing a coin) which eye receives which surgery (randomization). Randomization ensures that the study results are not falsified by unknown influencing factors, thus ensuring comparability of the surgical methods.

Neither you nor your doctor (who will examine your child) will know which eye has had which surgery before the study is completed. This is called blinding and is necessary to prevent any influence on the results. However, in an emergency, your doctor can always find out which eye has had which surgery. Only the surgeon knows this.

Are there any personal benefits from participating in the study?

There is no personal benefit to be expected from participating in the study. However, the results of the study may help other people in the future.

What are the risks associated with participating in the study?

Participation in the study is not associated with any additional medical risks. You and your child will be informed separately about the risks of the surgery.

What other options are there outside the study?

In clinical routine, both the 360° trabeculotomy and the probe trabeculotomy are performed. We are trying to find out which surgical method works better with this study.
Other surgical methods are the insertion of a drainage implant or the kryotherapy or photocoagulation of the ciliary body. However, these surgical methods are the second choice compared to trabeculotomy.

https://etic.med.tum.de

Are there any additional costs?

Neither you nor your health insurance company will incur any additional costs by participating in the study.

Was insurance taken out for this study?

During the study, the usual insurance cover of the Mainz University Medical Center applies. Insurance cover is provided if the doctor or another employee of the trial site is accused of culpable misconduct.

In this clinical study, all study participants are also insured under a volunteer and commuting accident insurance policy. The scope of the insurance cover can be found in the insurance documents that you will receive.

If you suspect that your child's health has been damaged or existing conditions have been exacerbated by participation in the clinical trial, you must inform the insurer immediately.

Name and address of the insurance company:	HDI Global SE<br>Proactiv Platz 1<br>40721 Hilden<br>phone: +49 (0) 211 7482-0<br>email: Christoph.Schmidt@hdi.global
Annual contract for volunteer and commuting accident insurance no:	76 307104 03017/03059

directly, if necessary, with the support of your study doctor, so as not to jeopardize your child's insurance cover. If your study doctor supports you in this, you will receive a copy of the notification. If you notify the insurer directly, please also inform your study doctor.

You must cooperate in clarifying the cause or extent of the damage and do everything possible to avert and minimize the damage.

A copy of the insurance confirmation and the insurance conditions will also be provided.

Information on data protection

In this study, the University Medical Center of Johannes Gutenberg University Mainz, represented by the Executive Board (Langenbeckstr. 1, 55131 Mainz, phone: +49 (0) 6131/17-0,

https://etic.med.tum.de

website: http://www.unimedizin-mainz.de/) is responsible for data processing. The legal basis for the processing is personal consent (Art. 6 para. 1 lit. A, Art. 9 para. 2 lit. A GDPR). The data will be treated confidentially at all times.

The data is collected exclusively for the purpose of this study described above and is only used within this framework.

The data collected also includes personal identifying data such as name, address, date of birth and sensitive personal health data.

The data that we process from your child is personal data in accordance with Art. 4 No. 1 -GDPR or special categories of personal data in the form of health data in accordance with Art. 4 No. 15 -GDPR-.

Specifically, we process the following data: Data collected as part of your child's medical history such as family history of glaucoma, date of birth and onset of disease etc.) or measured (highest intraocular pressure ever measured, current intraocular pressure, visual acuity, eye length, revision surgery and other ocular parameters).

We also collect other sensitive personal data. This includes your ethnic origin (country) and the degree to which you are related to each other.

All data that could be used to directly identify your child, e.g. their name or date of birth, is replaced by an identification code (pseudonymized). This makes it almost impossible for unauthorized persons to identify your child. Identification can only take place via the pseudonymization list. This is only accessible to the scientists involved.

The data is stored at the Interdisciplinary Center for Clinical Studies at the Mainz University Medical Center.

We only store the personal data for as long as is necessary for the above-mentioned purpose. The data will be deleted at the latest 10 years after discontinuation or termination of the study, unless statutory retention periods prevent this.

We do not transfer personal data to other institutions in Germany, the EU, to a third country outside the EU or to an international organization.

The data is passed on to the following institutions that are not directly involved in the treatment:

- The IZKS (Interdisciplinary Center for Clinical Studies) of the University Medical Center Mainz is creating a study database on behalf of the Eye Clinic in which the pseudonymized data of your child will be stored.
- The IMBEI (Institute for Medical Biometry, Epidemiology and Informatics) of the Mainz University Medical Center will analyze the pseudonymized data.

https://etic.med.tum.de

The data is published in anonymized form.

We do not transfer personal data to other institutions in Germany, the EU, to a third country outside the EU or to an international organization.

Consent to the processing of your and your child's data is voluntary. You can withdraw your consent at any time without giving reasons and without any disadvantages for you or your child. No more data will then be collected. This does not affect the lawfulness of the processing carried out on the basis of the consent until revocation.

In the event of revocation, you can request the deletion of the data collected. The data can also be used in anonymized form if you consent to this at the time of your revocation.

You have the right to receive information about the data, also in the form of a copy free of charge. In addition, you may request the rectification, blocking, restriction of processing or erasure and, where applicable, the portability of the data. You also have the right to object to the processing.

The personal data collected is not subject to decisions based solely on automated processing (e.g. profiling).

In these cases, please contact us if you have any further questions about data protection and the handling of data or in the event of revocation:

Prof. Dr. med. Esther M. Hoffmann
Department of Ophthalmology
University Medical Center Mainz
Langenbeckstraße 1
55131 Mainz
telephone: +496131/17 5150
email: ehoffman@uni-mainz.de

If you have any questions about data processing and compliance with data protection, please contact the Data Protection Officer of the Mainz University Medical Center:

Langenbeckstraße 1
55131 Mainz
phone: +49 (0) 6131/17-0
email: datenschutz@unimedizin-mainz.de

You also have the right to lodge a complaint with any data protection supervisory authority. You can find a list of the supervisory authorities in Germany at: https://www.bfdi.bund.de/DE/Infothek/Anschriften_Links/anschriften_links-node.html

https://etic.med.tum.de

You can contact the supervisory authority responsible for the Mainz University Medical Center, the State Commissioner for Data Protection and Freedom of Information, at

P.O. Box 30 40, 55020 Mainz
Hintere Bleiche 34, 55116 Mainz
phone: +49 (0) 6131 8920-0
fax: +49 (0) 6131 8920-299
email: poststelle@datenschutz.rlp.de
https://www.datenschutz.rlp.de

https://etic.med.tum.de

Director
Prof. Dr. med. Norbert Pfeiffer

Study center:
Department of Ophthtalmology
Mainz University Medical Center
Langenbeckstr. 1
55131 Mainz
phone: +49 (0) 6131 17-5150
email: glaukom-OP@unimedizin-mainz.de
Investigator:
Prof. Dr. med. Esther M. Hoffmann

Subject ID: _______________

Informed consent for the research project: Surgical success rates in pediatric glaucoma: Probe trabeculotomy versus microcatheter-assisted 360° trabeculotomy

I have been informed about the study by ____________________________. I have received and read the written information and declaration of consent for the above-mentioned study. I have been informed in detail in writing and verbally about the purpose and course of the study, the opportunities and risks of participation and the associated rights and obligations. I was also -comprehensively informed about the processing of my personal data in accordance with Art. 13 -GDPR. I had the opportunity to ask questions. These were answered satisfactorily and in full. In addition to the written information, the following points were discussed

____

____

____

My consent for my child

_______________________________________, born on _______________________,

participation in the study is voluntary. I have the right to withdraw this consent at any time without giving reasons and without any disadvantages for me or my child.

The processing and use of personal data for the above-mentioned study is carried out exclusively as described in the information on the study.

I have understood and agree to this,

1. that my personal data required for the purpose of the above-mentioned study (in particular health data and information on my child's ethnic origin) will be collected by the study doctor and recorded and processed in pseudonymized form, including on electronic data carriers;

2. that the study results will be published in an anonymous form that does not allow any conclusions to be drawn about my person;

3. that my data will be processed exclusively for the above-mentioned purposes and only by study staff or the named recipients or categories of recipients

We have received one copy of the information and consent. One copy remains at the test center.

Consent of the patient:

Patient: I give my consent to participate in the above-mentioned study.

____
Name of the patient

____
Place, date Signature **of the patient**

Consent of the legal guardian(s):

I hereby consent to my child taking part in the above-mentioned study.

____
Name of the **first legal guardian** in block capitals

____
Place, date Signature of the **first legal guardian**

____
Name of the **second legal guardian** in block capitals

____
Place, date Signature of the **second legal guardian**

If only one parent or legal guardian signs, the signature confirms that this is done in agreement with the other parent or that the person signing has sole custody:
Please comment accordingly by the sole custodian:

____
Name of the sole custodian in block capitals

____
Place, date Signature of the **sole custodian**

Witness/Interpreter

I was present throughout the process of informing the participant and confirm that the information about the aims and procedures of the study was adequately communicated, that the participant (or their legal representative) clearly understood the study and that consent to participate in the study was given voluntarily.

____
Name and qualification of the witness/interpreter in block capitals

____
Place, date Signature of the **witness/interpreter**

Informing doctor:

I have conducted the informed consent discussion and obtained the consent of the patient's legal guardians and the patient.

I hereby confirm that I have informed the above-mentioned legal guardians and the patient about the nature, purpose and foreseeable effects of the study. All questions have been answered and I have received a copy of the study information and informed consent form. The legal guardians and the patient have agreed to voluntary participation in the study with their signature.

____
Name of the informing investigator in block capitals

____
Place, date Signature of the **informing investigator**

Director
Prof. Dr. med. Norbert Pfeiffer

Study center:
Department of Ophthalmology
Mainz University Medical Center
Langenbeckstr. 1
55131 Mainz
phone: +49 (0) 6131 17-5150
email: glaukom-OP@unimedizin-mainz.de
Investigator:
Prof. Dr. med. Esther M. Hoffmann

Subject ID: _______________

Information for children (< 7 years): Surgical success rates in childhood glaucoma: Probe trabeculotomy versus microcatheter-based 360° trabeculotomy

Dear ,

we would like to ask you if you would like to take part in a research project and explain what this means. If you have any questions, you can ask them at any time. We will also explain the whole thing to your parents.

Perhaps your eyes sometimes hurt, water or your vision is blurred. This is because the pressure in your eyes is increased because the water in your eye cannot drain away properly. If we do nothing, you may not be able to see properly in the future. We would like to change this with a surgery. So that your eyes can continue to see well for a long time, the drainage channel into which the water in the eye can drain is cleared again.

https://etic.med.tum.de

You can imagine it like a gutter:

The purpose of the gutter is to collect rainwater that runs off the roof and drain it underground:

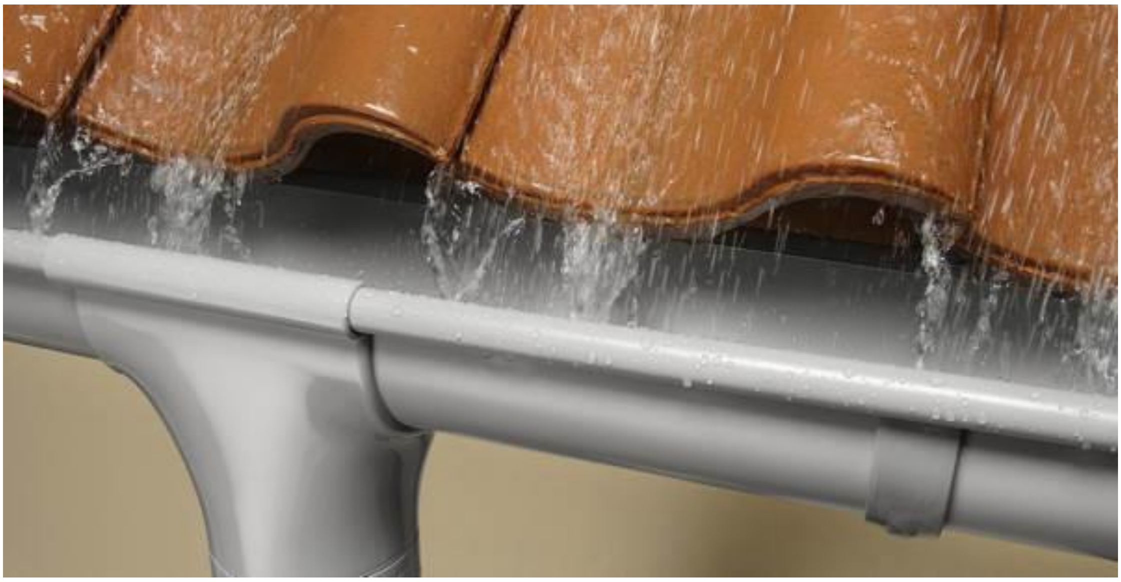

Altmeyer, H. and photos and graphics: Marley Germany (2013, 13.08.2013). "Protection against heavy rain." Retrieved 02.04.2024, 2024, from https://www.diy-info.de/files/massnahmen-gegen-regenwasser-schaeden.php.

Sometimes, however, leaves accumulate in the gutter so that it becomes blocked, and the water can no longer drain away properly:

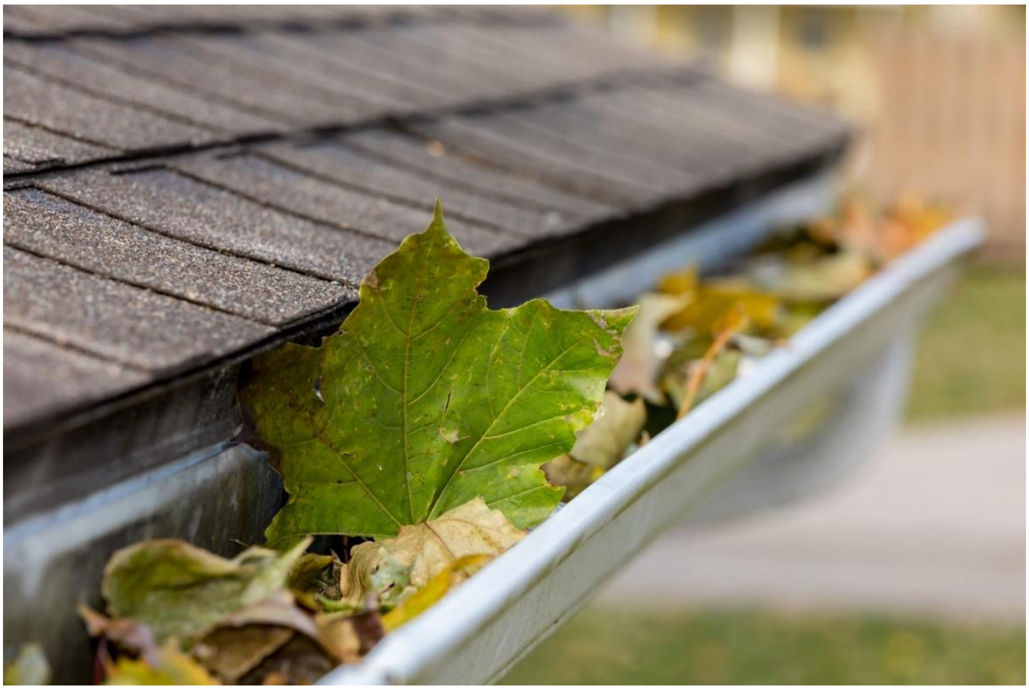

OBI editorial team (2022, 03.02.2022). "Cleaning the gutter." Retrieved 02.04.2024, 2024, from https://www.obi.de/magazin/bauen/aussenbereich/dachrinne-reinigen.

In the case of the gutter, the water simply overflows and anyone walking underneath gets soaking wet:

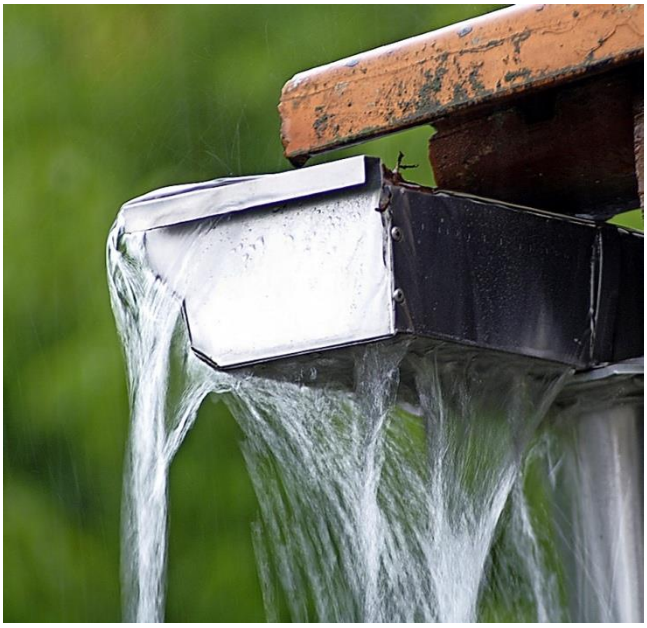

BZ-Redaktion and F. M. F.-. stock.adobe.com (2022, 04.03.2020). "Cleaning the gutter." Retrieved 02.04.2024, 2024, from https://www.badische-zeitung.de/damit-es-gut-laeuft.

The water that collects in your eye must be able to run off just like rainwater. There is a gutter that runs in a circle around your iris for this purpose. In your case, the gutter is blocked. However, as the eye is a closed sphere, the water cannot overflow. This is why the pressure in your eye rises sharply and your eye hurts and is reddened:

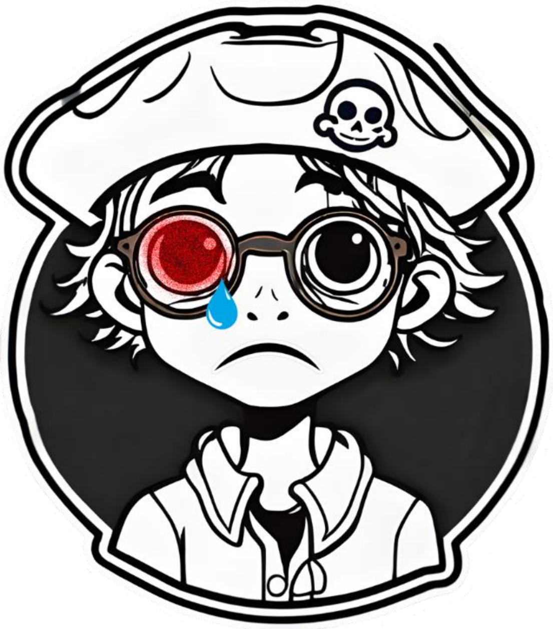

So that you can still see well, the gutter in your eye must be cleared again, as with the gutter on the roof that is blocked with leaves:

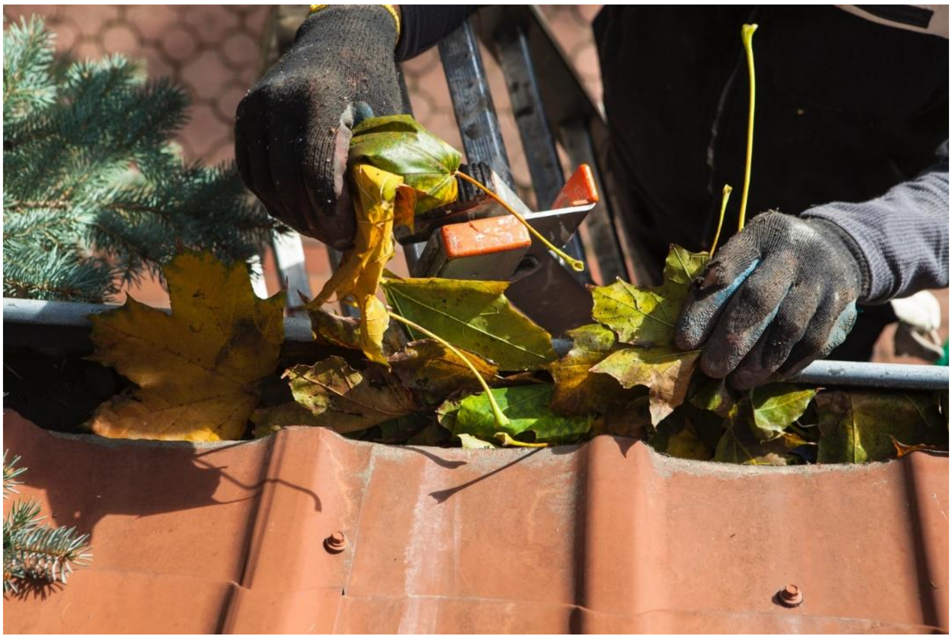

Source: OBI editorial team (2022, 03.02.2022). "Clean the gutter." Retrieved 02.04.2024, 2024, from https://www.obi.de/magazin/bauen/aussenbereich/dachrinne-reinigen.

This can be achieved with an eye surgery. There are two different types of surgery. Because the blocked eye channel is so rare, it has not yet been investigated which surgical method is better. We are trying to figure it out.

You don't have to be afraid of the surgery because you will be asleep and won't notice anything. After the surgery, you will have a bandage on your eye to protect it:

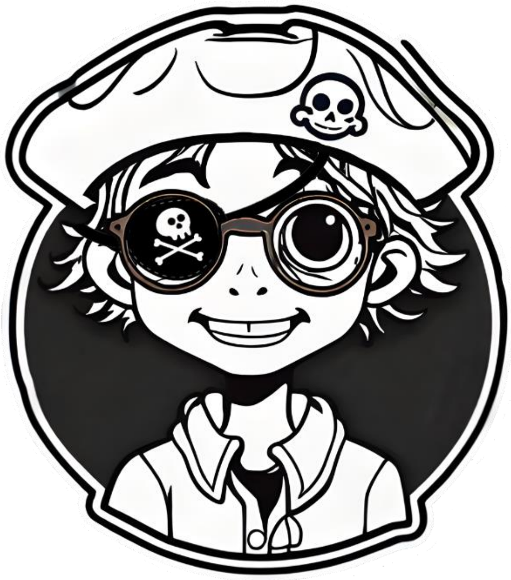

There is usually little to no pain. When the bandage is removed, you may have slightly blurred vision, but this will improve during the following days! Very importantly, if you feel anything strange after the surgery, tell your parents or your doctor immediately. This is very important so that we can help you properly.

If you do not wish to participate in the research project, we would still do the surgery, but both eyes would be treated with the same type of surgery.

In both cases, we would like to observe how your eyes develop. To do this, you will come to us regularly over the next 2 years.

If you have any further questions, please feel free to contact us. We will be happy to answer all your questions. Just give us a call: +49 (0) 6131-17 5150

Director
Prof. Dr. med. Norbert Pfeiffer

Study center:
Department of Ophthalmology
Mainz University Medical Center
Langenbeckstr. 1
55131 Mainz
phone: +49 (0) 6131 17-5150
email: glaukom-OP@unimedizin-mainz.de
Investigator:
Prof. Dr. med. Esther M. Hoffmann

Subject ID: _______________

Information for parents/guardians on the research project: Surgical success rates in childhood glaucoma: Probe trabeculotomy versus microcatheter-assisted 360° trabeculotomy

Dear parents,

Dear guardians,

We would like to ask you to allow your child to take part in a scientific study. In this information you will find everything you need to know about the study.

Please read this information carefully. Your doctor will talk to you about the study and answer your questions.

A total of 76 patients will be included. It is planned to conduct the study at 3 centers (Mainz, Cologne, Leuven/Belgium).

This study is planned, conducted and monitored by the Department of Ophthalmology of the University Medical Center Mainz. It is funded by the German Research Foundation.

The study was submitted to the responsible ethics committee. It raised no objections.

Your decision to allow your child to participate in the study is voluntary. You can also terminate your child's participation at any time during the course of the study (verbally, in writing or in text form). You do not have to justify this. This will not result in any disadvantages for you or your child. In particular, your decision will not result in any disadvantages for your child's medical treatment or the relationship with the doctor treating him/her.

https://etic.med.tum.de

If you have any further questions about the study now or later, please feel free to contact us. Please contact Prof. Dr. Esther M. Hoffmann (email: glaukom-op@unimedizin-mainz.de, telephone: +49 (0) 6131/17 5150).

Why is this study being conducted?

Your daughter/son is suffering from childhood glaucoma. The cause of such glaucoma is usually incomplete development of the eye, in which the outflow of water in the eye (the so-called aqueous humor) does not function normally. As a result, the intraocular pressure rises sharply and the optic nerve is damaged. This is usually accompanied by clouding of the cornea and growth of the eyeball. Affected children often suffer from sensitivity to light, tearing, eyelid spasms and rubbing their eyes.
Glaucoma only occurs very rarely at such an early age. Because only a few children are affected, childhood glaucoma and its treatment are not well researched.
Treatment usually requires a surgery to prevent blindness. The surgery of first choice is trabeculotomy, in which the outflow pathway of the aqueous humor is restored. The collecting duct that surrounds the anterior chamber of the eye and into which the aqueous humor normally drains is exposed and opened towards the anterior chamber of the eye. There are 2 different surgical methods for this: with the help of an illuminated silicone tube, the canal can be opened completely over 360° (360° trabeculotomy, see Figure 1). Metal probes can also be used to open the canal by 90 to 120° (probe trabeculotomy, see Figure 2).

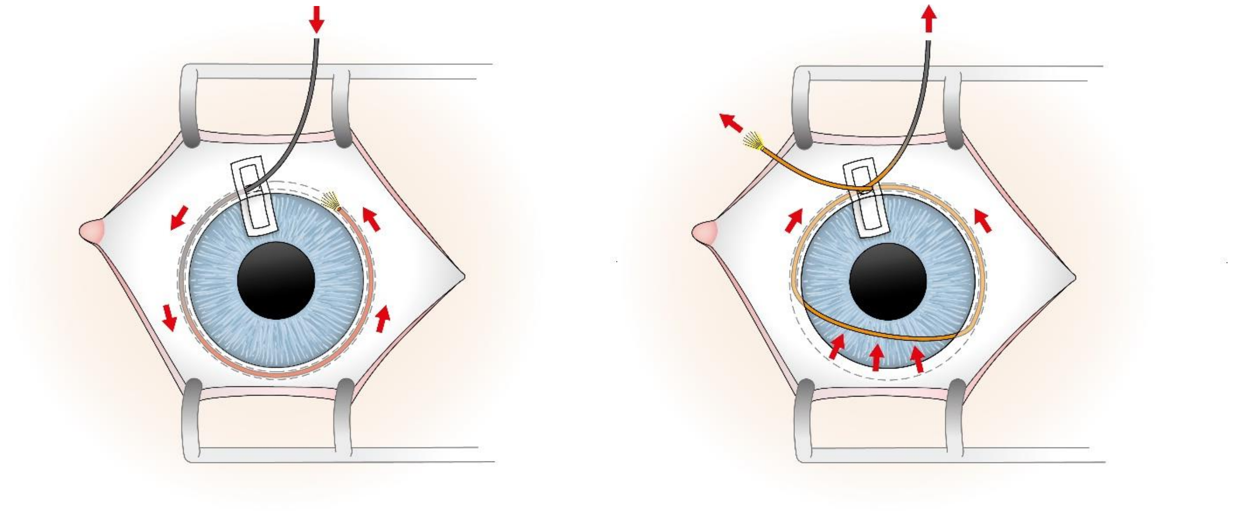

Figure 1 Complete opening using 360° trabeculotomy
Left: A thin silicone tube ("microcatheter") is threaded into the outflow channel of the eye.
Right: The ends of the silicone tube are pulled together so that the outflow channel opens towards the anterior chamber of the eye. The silicone tube then appears in the anterior chamber of the eye. The eye fluid can now drain away again.

https://etic.med.tum.de

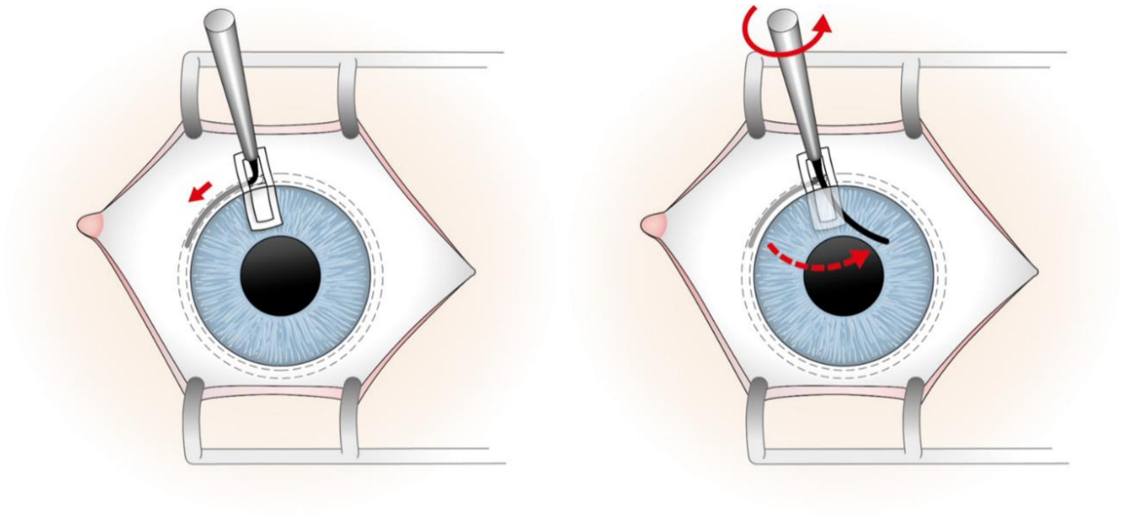

Figure 2 Partial opening by means of probe trabeculotomy (90-120°)
Left: A thin metal rod ("probe") is inserted into the outflow tract.
Right: The tip of the probe is turned towards the pupil so that the outflow tract opens a short distance towards the anterior chamber of the eye. The probe then appears in the anterior chamber of the eye. The aqueous humor can now flow out again. This is carried out in both the right and left direction.

Our clinical experience to date with both surgical techniques has shown the following possible advantages and disadvantages of each method:

The advantage of the probe trabeculotomy is the slightly shorter (approx. 2-3 minutes) surgery time and the slightly less bleeding from the blood-filled Schlemm's canal, as only 120° is opened.

The advantage of the 360° trabeculotomy is the circular opening of the canal and the assumption of greater pressure reduction. The disadvantage is the increased bleeding (hyphaema) and therefore possibly faster scarring than with the conventional method.

As these assumptions have not yet been scientifically confirmed, we hope that this study will enable us to better assess the advantages and disadvantages.

In this study we would like to find out which type of trabeculotomy is more successful. For this purpose, the intraocular pressure 2 years after surgery will be compared. One eye will receive a 360° trabeculotomy, the other a probe trabeculotomy. This will ensure optimal comparability between the two surgical procedures.

How does the study work?

The study is expected to last 2 years for each participant.

If possible, both eyes are treated on in one session. The aim of this procedure is to save on anesthesia and allow both eyes to be relieved at the same time. If this is not possible, the

https://etic.med.tum.de

second eye can also be treated on at a later date.
On the first day after the surgery, a visit is made as part of the inpatient stay. Otherwise, 6 follow-up examinations are planned at the clinic. They take place after 4 weeks, 3 months, 6 months, 12 months, 18 months and 24 months. Normally, awake examinations are sufficient; however, an anesthetic examination may be necessary if the examination conditions are poor and if it is suspected that the intraocular pressure has not been reduced sufficiently. Two hours should be allowed for an examination on an awake child. For an examination under anesthesia, 2 days should be planned and an inpatient stay is necessary. The follow-up examinations correspond to the normal rhythm and the normal examination spectrum of the clinical routine, so that no additional examinations and appointments will be necessary. All examinations would be carried out in the same way if your child had a trabeculotomy outside of the study.

As each child receives both surgical methods that are compared in the study, it is determined at random (like tossing a coin) which eye receives which surgery (randomization). Randomization ensures that the study results are not falsified by unknown influencing factors, thus ensuring comparability of the surgical methods.

Neither you nor your doctor (who will examine your child) will know which eye has had which surgery before the study is completed. This is called blinding and is necessary to prevent any influence on the results. However, in an emergency, your doctor can always find out which eye has had which surgery. Only the surgeon knows this.

Are there any personal benefits from participating in the study?

There is no personal benefit to be expected from participating in the study. However, the results of the study may help other people in the future.

What are the risks associated with participating in the study?

Participation in the study is not associated with any additional medical risks. You and your child will be informed separately about the risks of the surgery.

What other options are there outside the study?

In clinical routine, both the 360° trabeculotomy and the probe trabeculotomy are performed. We are trying to find out which surgical method works better with this study.
Other surgical methods are the insertion of a drainage implant or the kryotherapy or photocoagulation of the ciliary body. However, these surgical methods are the second choice compared to trabeculotomy.

https://etic.med.tum.de

Are there any additional costs?

Neither you nor your health insurance company will incur any additional costs by participating in the study.

Was insurance taken out for this study?

During the study, the usual insurance cover of the Mainz University Medical Center applies. Insurance cover is provided if the doctor or another employee of the trial site is accused of culpable misconduct.

In this clinical study, all study participants are also insured under a volunteer and commuting accident insurance policy. The scope of the insurance cover can be found in the insurance documents that you will receive.

If you suspect that your child's health has been damaged or existing conditions have been exacerbated by participation in the clinical trial, you must inform the insurer immediately.

Name and address of the insurance company:	HDI Global SE<br>Proactiv-Platz 1<br>40721 Hilden<br>phone: +49 (0) 211 7482-0<br>email: Christoph.Schmidt@hdi.global
Annual contract for volunteer and commuting accident insurance no:	76 307104 03017/03059

directly, if necessary, with the support of your study doctor, so as not to jeopardize your child's insurance cover. If your study doctor supports you in this, you will receive a copy of the notification. If you notify the insurer directly, please also inform your study doctor.

You must cooperate in clarifying the cause or extent of the damage and do everything possible to avert and minimize the damage.

A copy of the insurance confirmation and the insurance conditions will also be provided.

Information on data protection

In this study, the University Medical Center of Johannes Gutenberg University Mainz, represented by the Executive Board (Langenbeckstr. 1, 55131 Mainz, phone +49 (0)6131/17-0, website: http://www.unimedizin-mainz.de/) is responsible for data processing. The legal basis for the processing is personal consent (Art. 6 para. 1 lit. A, Art. 9 para. 2 lit. A GDPR). The data will be treated confidentially at all times.

The data is collected exclusively for the purpose of this study described above and is only used within this framework.

The data collected also includes personal identifying data such as name, address, date of birth and sensitive personal health data.

The data that we process from your child is personal data in accordance with Art. 4 No. 1 -GDPR or special categories of personal data in the form of health data in accordance with Art. 4 No. 15 -GDPR-.

Specifically, we process the following data: Data collected as part of your child's medical history such as family history of glaucoma, date of birth and onset of disease etc.) or measured (highest intraocular pressure ever measured, current intraocular pressure, visual acuity, eye length, revision surgery and other ocular parameters).

We also collect other sensitive personal data. This includes your ethnic origin (country) and the degree to which you are related to each other.

All data that could be used to directly identify your child, e.g. their name or date of birth, is replaced by an identification code (pseudonymized). This makes it almost impossible for unauthorized persons to identify your child. Identification can only take place via the pseudonymization list. This is only accessible to the scientists involved.

The data is stored at the Interdisciplinary Center for Clinical Studies at the Mainz University Medical Center.

We only store the personal data for as long as is necessary for the above-mentioned purpose. The data will be deleted at the latest 10 years after discontinuation or termination of the study, unless statutory retention periods prevent this.

We do not transfer personal data to other institutions in Germany, the EU, to a third country outside the EU or to an international organization.

The data is passed on to the following institutions that are not directly involved in the treatment:

https://etic.med.tum.de

- The IZKS (Interdisciplinary Center for Clinical Studies) of the University Medical Center Mainz is creating a study database on behalf of the Eye Clinic in which the pseudonymized data of your child will be stored.
- The IMBEI (Institute for Medical Biometry, Epidemiology and Informatics) of the Mainz University Medical Center will analyze the pseudonymized data.

The data is published in anonymized form.

We do not transfer personal data to other institutions in Germany, the EU, to a third country outside the EU or to an international organization.

Consent to the processing of your and your child's data is voluntary. You can withdraw your consent at any time without giving reasons and without any disadvantages for you or your child. No more data will then be collected. This does not affect the lawfulness of the processing carried out on the basis of the consent until revocation.

In the event of revocation, you can request the deletion of the data collected. The data can also be used in anonymized form if you consent to this at the time of your revocation.

You have the right to receive information about the data, also in the form of a copy free of charge. In addition, you may request the rectification, blocking, restriction of processing or erasure and, where applicable, the portability of the data. You also have the right to object to the processing.

The personal data collected is not subject to decisions based solely on automated processing (e.g. profiling).

In these cases, please contact us if you have any further questions about data protection and the handling of data or in the event of revocation:

Prof. Dr. med. Esther M. Hoffmann
Department of Ophthalmology
University Medical Center Mainz
Langenbeckstraße 1
55131 Mainz
phone: 06131/17 5150
email: ehoffman@uni-mainz.de

If you have any questions about data processing and compliance with data protection, please contact the Data Protection Officer of the Mainz University Medical Center:

Langenbeckstraße 1
55131 Mainz
phone: +49 (0) 6131/17-0
email: datenschutz@unimedizin-mainz.de

https://etic.med.tum.de

You also have the right to lodge a complaint with any data protection supervisory authority. You can find a list of the supervisory authorities in Germany at: https://www.bfdi.bund.de/DE/Infothek/Anschriften_Links/anschriften_links-node.html

You can contact the supervisory authority responsible for the Mainz University Medical Center, the State Commissioner for Data Protection and Freedom of Information, at

P.O. Box 30 40, 55020 Mainz
Hintere Bleiche 34, 55116 Mainz
phone: +49 (0) 6131 8920-0
fax: +49 (0) 6131 8920-299
email: poststelle@datenschutz.rlp.de
https://www.datenschutz.rlp.de

Director
Prof. Dr. med. Norbert Pfeiffer

Study center:
Department of Ophthalmology
Mainz University Medical Center
Langenbeckstr. 1
55131 Mainz
phone: +49 (0) 6131 17-5150
email: glaukom-OP@unimedizin-mainz.de
Investigator:
Prof. Dr. med. Esther M. Hoffmann

Subject ID: _______________

Informed consent for the research project: Surgical success rates in pediatric glaucoma: Probe trabeculotomy versus microcatheter-assisted 360° trabeculotomy

I have been informed about the study by ____________________________. I have received and read the written information and declaration of consent for the above-mentioned study. I have been informed in detail in writing and verbally about the purpose and course of the study, the opportunities and risks of participation and the associated rights and obligations. I was also -comprehensively informed about the processing of my personal data in accordance with Art. 13 -GDPR. I had the opportunity to ask questions. These were answered satisfactorily and in full. In addition to the written information, the following points were discussed

____

____

____

My consent for my child

_______________________________________, born on _______________________,

participation in the study is voluntary. I have the right to withdraw this consent at any time without giving reasons and without any disadvantages for me or my child.

The processing and use of personal data for the above-mentioned study is carried out exclusively as described in the information on the study.

I have understood and agree to this,

1. that my personal data required for the purpose of the above-mentioned study (in particular health data and information on my child's ethnic origin) will be collected by the study doctor and recorded and processed in pseudonymized form, including on electronic data carriers;

2. that the study results will be published in an anonymous form that does not allow any conclusions to be drawn about my person;

3. that my data will be processed exclusively for the above-mentioned purposes and only by study staff or the named recipients or categories of recipients

We have received one copy of the information and consent. One copy remains at the test center.

Consent of the patient:

Patient: I give my consent to participate in the above-mentioned study.

____
Name of the patient

____
Place, date Signature **of the patient**

Consent of the legal guardian(s):

I hereby consent to my child taking part in the above-mentioned study.

____
Name of the **first legal guardian** in block capitals

____
Place, date Signature of the **first legal guardian**

https://etic.med.tum.de

____
Name of the **second legal guardian** in block capitals

____
Place, date Signature of the **second legal guardian**

If only one parent or legal guardian signs, the signature confirms that this is done in agreement with the other parent or that the person signing has sole custody:
Please comment accordingly by the sole custodian:

____
Name of the sole custodian in block capitals

____
Place, date Signature of the **sole custodian**

Witness/Interpreter

I was present throughout the process of informing the participant and confirm that the information about the aims and procedures of the study was adequately communicated, that the participant (or their legal representative) clearly understood the study and that consent to participate in the study was given voluntarily.

____
Name and qualification of the witness/interpreter in block capitals

____
Place, date Signature of the **witness/interpreter**

Informing physician:

I have conducted the informed consent discussion and obtained the consent of the patient's legal guardians and the patient.

I hereby confirm that I have informed the above-mentioned legal guardians and the patient about the nature, purpose and foreseeable effects of the study. All questions have been answered and I have received a copy of the study information and informed consent form. The legal guardians and the patient have agreed to voluntary participation in the study with their signature.

____
Name of the informing investigator in block capitals

____
Place, date Signature of the **informing investigator**

https://etic.med.tum.de
